# Curve walking gait analysis using depth camera and machine learning for detecting cognitive impairment

**DOI:** 10.3389/fneur.2026.1777635

**Published:** 2026-09-29

**Authors:** Mahmoud Seifallahi, James E. Galvin, Behnaz Ghoraani

**Affiliations:** 1 Department of Computer and Electrical Engineering and Computer Science, Florida Atlantic University, Boca Raton, FL, United States; 2 Comprehensive Center for Brain Health, Department of Neurology, University of Miami, Boca Raton, FL, United States

**Keywords:** Alzheimer's disease (AD), cognitive impairment (CI), curve walking, dual-task, Kinect v.2 camera, machine learning, mild cognitive impairment (MCI)

## Abstract

**Introduction:**

Early detection of cognitive impairment (CI), including MCI and Alzheimer's disease (AD), remains limited by the invasiveness and cost of current diagnostic tools. While gait analysis is a promising alternative, most studies focus on straight-path walking, which may miss subtle motor-cognitive deficits. We hypothesized that curve walking—especially under dual-task (DT) conditions—would better expose early CI-related changes. Combined with machine learning, this approach offers a low-cost, non-invasive tool to distinguish healthy controls (HC), MCI, and AD.

**Methods:**

Seventy-three older adults (HC, MCI due to Alzheimer's disease, and mild AD) performed curve walking along a 10-m oval path under single-task (ST) and DT conditions (counting backward from 100 by ones) while 3D body joint motion was captured using a Kinect v.2 depth camera. Comprehensive macro- and micro-level gait markers were extracted using signal processing and statistical analysis, followed by gait marker selection and machine learning-based classification of CI vs. HC and AD vs. MCI.

**Results:**

Out of 156 extracted gait markers, statistical analysis identified 22 ST and 34 DT markers—including macro, micro-temporal, spatial, and symmetry markers—as selected significantly discriminative markers. CI participants showed slower, less stable gait, with greater variability and asymmetry, particularly under DT conditions. Machine learning models combining ST and DT markers achieved 89.04% accuracy for CI vs. HC and 93.02% for AD vs. MCI, confirming that curved walking under dual-task conditions provides richer discriminative gait information than single-task curved walking alone, and supporting curved dual-task gait analysis as a promising paradigm for early cognitive impairment detection and staging.

**Discussion:**

Curve walking under cognitive load revealed subtle gait deficits linked to CI, enabling accurate, low-cost, and non-invasive detection and staging using depth camera data and machine learning—supporting its potential as a screening approach for supervised clinical and community settings, subject to validation in less-controlled environments.

## 1 Introduction

Alzheimer's disease (AD) and related disorders are the most prevalent causes of dementia in older adults and are characterized by progressive cognitive decline that interferes with daily functioning and independence (1–3). Despite their widespread impact, cognitive assessments are not routinely administered—only 16% of older adults undergo regular screening for cognitive impairment (3). Early symptoms are often misattributed to normal aging (4), and formal diagnoses typically occur only when cognitive deficits become clearly apparent (5, 6). As a result, many individuals are diagnosed too late to benefit from emerging disease-modifying therapies, which are most effective when administered during the early stages of the disease (7).

Timely detection of cognitive decline is critical, and research has increasingly focused on identifying Mild Cognitive Impairment (MCI) as an early and clinically meaningful stage of Alzheimer's disease and related disorders (ADRD). MCI represents a clinically heterogeneous condition in which individuals exhibit measurable cognitive decline beyond what is expected for normal aging, yet without significant loss of functional independence (8–10). While not all individuals with MCI progress to dementia, those affected carry a substantially elevated risk—particularly to Alzheimer's disease—and MCI is recognized as a heterogeneous condition that may reflect the prodromal stage of various dementia subtypes, including but not limited to Alzheimer's disease, vascular dementia, and Lewy body dementia (11). When MCI is due to underlying Alzheimer's pathology, affected individuals show biomarker abnormalities and face an elevated risk of progressing to Alzheimer's dementia, with annual conversion rates ranging from 10 to 15% (12). However, because early-stage symptoms are subtle, up to 80% of MCI cases remain undiagnosed, further contributing to treatment delays (13, 14).

Current diagnostic approaches for MCI and AD often involve neuroimaging (e.g., MRI, PET), cerebrospinal fluid analysis, blood-based biomarkers, and extensive neuropsychological testing (15, 16). While these methods provide high diagnostic accuracy, they are also expensive, invasive, and time-consuming, requiring specialized clinical expertise. As a result, they are generally reserved for individuals already exhibiting moderate cognitive symptoms, limiting their value for large-scale screening and early detection. There is a growing need for non-invasive, low-cost, and scalable tools that can assess cognitive function in community settings and serve as a first step toward more detailed diagnostic evaluation.

The neurophysiological basis for gait changes in AD and MCI is fundamentally distinct from that of movement disorders such as Parkinson's disease, where dopaminergic loss in the basal ganglia directly impairs motor automaticity. In AD and MCI, gait alterations arise through at least three converging mechanisms. First, the hippocampus and entorhinal cortex—among the earliest and most severely affected structures in AD, exhibiting neurodegeneration as early as Braak stage I–II—are critically involved in spatial navigation and path integration (17, 18). Because curved walking requires continuous spatial recalibration, directional orientation, and dynamic postural adjustment along a non-linear trajectory, it places direct demands on precisely these compromised circuits, making it uniquely sensitive to early AD pathology compared with rhythmic straight-path walking (19). Second, well-documented dysfunction of the frontoparietal network in MCI and AD impairs executive function, selective attention, and cognitive-motor coordination (20); these higher-order resources are essential for supervising non-automatic gait, particularly under cognitively demanding conditions (21). Third, white-matter changes and cortical atrophy in premotor and supplementary motor areas—measurable even in early MCI—further undermine the neural substrate for adaptive gait control (22). Together, these changes render gait less automatic in individuals with MCI and AD, so that walking competes more heavily with concurrent cognitive tasks for shared attentional resources (23). This is the neurophysiological basis for the disproportionate gait deterioration observed during dual-task conditions in cognitively impaired individuals (24). Curved walking is therefore especially sensitive to these deficits because, unlike rhythmic straight-path walking, it cannot be performed automatically—it demands continuous spatial recalibration, dynamic balance, and executive motor planning, precisely the functions subserved by the hippocampal-entorhinal and frontoparietal systems compromised in early AD.

An emerging solution is the use of gait analysis—particularly under dual-task (DT) conditions—as an indicator of cognitive decline. Gait is increasingly recognized as a “motor-cognitive” function, and impairments in gait dynamics often precede overt cognitive symptoms (25–28). DT walking assessments, in which individuals perform a cognitive task (e.g., counting backward) while walking, can expose subtle motor-cognitive deficits not evident in single-task (ST) walking (29). However, most existing studies rely on *straight-path walking*, which is repetitive and offers limited variability because of being a rhythmic action (5, 6). By contrast, *curve walking*, which involves continuous turning and requires greater postural control and cognitive engagement, may offer promising sensitivity for detecting early cognitive impairment, with this sensitivity potentially increased further when a concurrent cognitive task is added.

Despite its potential, curve walking has been underexplored. Prior research has primarily focused on brief turning events embedded in straight walking tasks or in the Timed Up and Go (TUG) test. Some studies have shown that turning velocity and related markers can differentiate MCI from healthy controls (30), and that turning-based metrics outperform straight-walking features when measured using depth cameras or smartphone sensors (31, 32). One major limitation is that many earlier systems rely on pressure-sensitive mats or space-intensive setups, which are not suited for continuous, curved-path gait assessment. These limitations have restricted the use of curve walking in large-scale cognitive screening.

Unlike straight-path walking, which is largely rhythmic and automatic, curved walking imposes substantially greater demands on postural control, spatial navigation, and cognitive engagement. From a biomechanical perspective, walking along a curved trajectory requires continuous asymmetric adjustment of stride length and stance time between the inner and outer legs, active trunk rotation, and dynamic maintenance of balance against centripetal forces—none of which are required in straight-line locomotion (33). Curved walking is therefore inherently non-automatic, requiring predictive control of the planned trajectory and continuous recalibration of body orientation in space (34). Turning and curved-path walking constitute a substantial portion of real-world locomotion—accounting for 8%–50% of all walking steps during daily activities (35)—making curved-path assessments ecologically valid and clinically relevant. The cognitive demands of curved walking are particularly meaningful for early CI detection: because curved walking cannot be performed by relying on automatic, rhythmic motor patterns, it continuously engages executive function, spatial planning, and attentional resources that are compromised early in MCI and AD (36). Prior studies have shown that turning velocity and curved-path gait metrics are more sensitive to cognitive status than straight-path equivalents (31), and that turning-based deficits can differentiate MCI from healthy controls even when straight-walking markers do not (37). Our own prior work in older adults with MCI confirmed that curved-path gait analysis identifies more and stronger group differences than straight-path walking (38).

To address this gap, we developed a novel Kinect-based system that enables continuous, full-body, markerless gait capture during curved-path walking. This technology allows for detailed analysis of macro- and micro-level gait markers—including symmetry, variability, and sub-phase timing—under both ST and DT conditions. Our prior work showed that ST oval-path walking captured with this system outperformed straight walking in distinguishing MCI from healthy controls (38). However, that study did not evaluate DT performance or assess individuals across the full cognitive spectrum (i.e., HC, MCI, and mild AD), which are addressed in the current work.

In this study, we build on that foundation and propose a non-invasive, accessible, and single camera-based gait assessment system to support early detection of MCI and AD. Participants perform curve walking along a defined oval path under both ST and DT conditions, while a Kinect v.2 depth camera captures body's joint movement data. Kinematic gait markers and DT costs are extracted from the recorded signals and analyzed using machine learning algorithms. Our classification strategy reflects clinical priorities by first distinguishing individuals with cognitive impairment (CI; encompassing both MCI and mild AD) from HC, and subsequently differentiating MCI from mild AD. Curve walking, due to its higher cognitive and balance demands (33, 37), is hypothesized to reveal more discriminative gait markers than straight walking. Our approach is cost-effective and space-efficient, making it suitable for supervised clinical and community settings such as memory clinics, geriatric offices and community centers; extension to less-controlled environments such as participants' homes would require further validation. Importantly, we demonstrate that combining markers from both ST and DT curve walking leads to highly accurate automatic classification of cognitive status, positioning this method as a promising candidate for early AD screening.

## 2 Materials and methods

### 2.1 Study population

Seventy-three participants aged 60 years or older were recruited for this study, including 30 HC, 25 individuals with MCI, and 18 with mild AD. Participants with MCI and mild AD were enrolled from the Iran Dementia and Alzheimer's Association (IDAA) after undergoing comprehensive diagnostic evaluations, including neuropsychological testing, magnetic resonance imaging (MRI), and blood analyses. All participants with MCI were clinically diagnosed as MCI due to Alzheimer's disease following the NIA-AA criteria (10). Healthy controls were recruited both from IDAA's dementia prevention program—following routine diagnostic screening—and from community volunteers. All participants were physically independent and had no history of stroke or major orthopedic interventions (e.g., hip or knee surgery) that could interfere with normal gait patterns. Each participant completed clinical assessments, including the Persian versions of the Mini-Mental State Examination (MMSE), Montreal Cognitive Assessment (MoCA), and Geriatric Depression Scale (GDS). GDS scores were interpreted as follows: 0–4 = normal, 5–9 = mild depression, 10–11 = moderate depression, and 12–15 = severe depression. Participants with severe depression (GDS > 11) were excluded from the study. This study was approved by the Ethics Committee of Semnan University of Medical Sciences in Iran (IRB approval number: IR.SEMUMS.REC.1398.237), and all procedures were conducted in accordance with the Declaration of Helsinki. Written informed consent was obtained from all participants, who were informed of their right to withdraw from the study at any time. Participant recruitment, clinical diagnosis, and gait recordings were performed at the Iran Dementia and Alzheimer's Association (IDAA). Gait data were collected by M. Seifallahi during his appointment as a research associate at IDAA, prior to joining Florida Atlantic University (FAU). The 3D pose signals extracted from the Kinect recordings were subsequently transferred as de-identified data and used for all signal processing, statistical analysis, and machine-learning development at FAU.

Table 1 summarizes the demographic and clinical characteristics of the participants. While no significant differences in age were observed across groups, significant group differences (*p* < 0.05) were found in gender distribution, body mass index (BMI), years of education, and GDS scores. Notably, HC and MCI participants had GDS scores in the normal range, whereas some participants with mild AD exhibited mild to moderate depression. As expected, participants with MCI and mild AD had significantly lower MMSE and MoCA scores compared to HC participants. To account for group differences in gender, BMI, education, and GDS, these variables were later included as covariates in the analysis using Analysis of Covariance (ANCOVA) to control for potential confounding effects (39).

**Table 1:** Demographic and clinical information of participants.

Characteristic	HC (*N* = 30)	MCI (*N* = 25)	Mild AD (*N* = 18)	*p*-value
Age (years)	68.33 ± 2.15	69.76 ± 6.45	72.61± 9.20	0.054
Female gender, *N* (%)	18 (60)^a^	9 (76)^b^	5 (27.78)^a, b^	0.007^*^
BMI (kg/*m*^2^)	24.51 ± 2.67^a^	26.67 ± 2.62^a^	25.58 ± 3.75	0.008^*^
Years of education	13.53 ± 3.05^a^	11.56 ± 3.00^a^	11.94 ± 5.63	0.027^*^
MMSE	28.50 ± 1.17^a, b^	25.60 ± 1.29^a^	23.38 ± 2.00^b^	< 0.001^*^
MoCA	27.13 ± 2.05^a, b^	22.76 ± 1.69^a^	21.33 ± 2.70^b^	< 0.001^*^
GDS	1.43 ± 1.33^a, b^	3.52 ± 1.29^a^	6.39 ± 4.22^b^	< 0.001^*^

Mean ± standard deviation was shown. *N* = number of participants; HC, healthy cognitive control group; MCI, mild cognitive impairment; AD, Alzheimer's disease; BMI, body mass index; MMSE, mini-mental state examination (maximum score, 30); MoCA, Montreal cognitive assessment (maximum score, 30); GDS, geriatric depression scale (maximum score, 15). The gait feature analysis was adjusted for the confounding variables of BMI, education, and GDS. ^a, b^ Same letters mean significant differences between groups (*p*-value < 0.05). ^*^ shows the significant difference.

### 2.2 Gait measurements

Participants performed ST and DT curve walking along a 10-meter oval path. The 10-meter walking length was selected to be consistent with standard clinical gait tests, which are commonly performed on a straight path (40). Each participant was allowed up to three practice trials to become familiar with the walking task. In the ST condition, participants walked at a comfortable, self-selected pace without any added cognitive load. In the DT condition, they repeated the same walking task while counting backward from 100 by ones. All assessments were performed independently, without any physical assistance from the research or clinical staff. Participants were allowed to rest for up to five minutes between trials, if needed. To accommodate individual preferences and minimize potential anxiety, participants were given the option to choose their walking direction—either clockwise or counterclockwise—thereby enhancing the ecological validity of the recordings. A review of the recorded data revealed that all 73 participants spontaneously elected to walk in the clockwise direction, resulting in a directionally uniform dataset. This eliminates direction as a potential confound of gait measurements or classification performance in the present cohort and is consistent with the population-level rightward locomotor bias documented in healthy adults (41).

Gait movement data were recorded using a Kinect v.2 depth camera, which was mounted on a tripod and positioned on the right side of the oval path. The Kinect system captured motion data from 25 body joints (42, 43). These joint signals were acquired through a custom Graphical User Interface (GUI) developed in MATLAB software. Figure 1 illustrates the recording setup and sample outputs. Figure 1B shows representative skeletal models constructed from the 25-joint coordinates, along with the corresponding RGB images captured during curve walking. The use of joint coordinate data offers a significant privacy advantage, as it eliminates the need for identifiable RGB video footage during data analysis.

**Figure 1:**
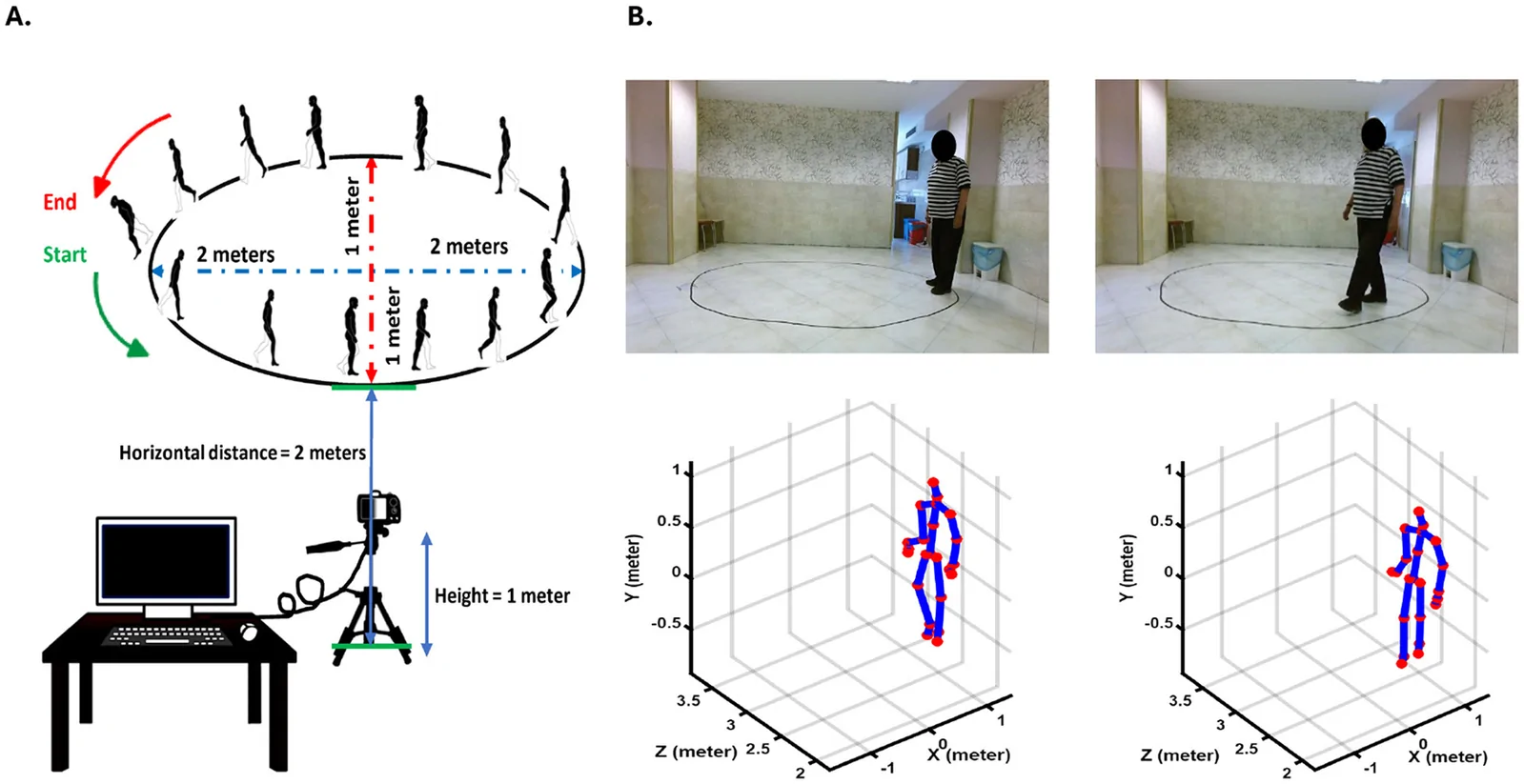
**(A)** Recording tools and setup. **(B)** Sample recorded data and their extracted skeletal data via depth camera of Kinect v.2.

### 2.3 Data processing

#### 2.3.1 Single and dual-task gait markers

A custom data processing pipeline was developed in MATLAB to extract a comprehensive set of kinematic gait markers from the Kinect v.2 recordings, following the methodology established in our prior work (44, 45). The extracted markers included both macro- and micro-level gait markers. Macro gait markers consisted of total walking time, average walking velocity, number of steps, and cadence. Micro gait markers were further categorized into temporal, spatial, and spatiotemporal domains. For each micro gait marker, we computed multiple descriptive statistics, including the mean, median, and variability. For gait step and stride-based measures, additional metrics such as symmetry and regularity were also calculated to capture more nuanced aspects of gait control.

To evaluate the impact of cognitive load on gait performance, we computed the cost of dual-tasking for each marker, hereafter referred to as Dual-Task Cost (DTC) and defined as the percentage change from the ST to the DT condition, computed as DTC = (DT − ST)/ST × 100. This approach enabled us to capture both absolute gait performance under cognitive interference and the relative degradation due to added cognitive demand. In total, 156 gait markers were derived per participant, comprising 52 markers from the ST condition and 104 from the DT condition, which included both direct DT values and DT cost measures. A complete list of all extracted gait markers, their values, and p-values of the comparison between study groups is provided in Table 2, Supplementary Tables S1, S2.

**Table 2:** Comparison of extracted gait markers from curve walking between study groups (CI vs. HC and AD vs. MCI) based on reported *p*-value in single and dual task conditions.

		***p*** **-value**					
Feature		Single-task		Dual-task		Dual-task cost	
Type	Name	CI vs. HC	AD vs. MCI	CI vs. HC	AD vs. MCI	CI vs. HC	AD vs. MCI
Macro	Duration (s)	< 0.001^*^	< 0.001^*^	< 0.001^*^	< 0.001^*^	< 0.001^*^	0.004^*^
	Velocity (cm/s)	< 0.001^*^	< 0.001^*^	< 0.001^*^	0.002^*^	0.496	0.171
	Step number	0.009^*^	< 0.001^*^	< 0.001^*^	< 0.001^*^	< 0.001^*^	< 0.001^*^
	Cadence	< 0.001^*^	0.040^*^	< 0.001^*^	0.043^*^	0.810	0.713
Micro temporal	Stance T. me (s)	< 0.001^*^	< 0.001^*^	< 0.001^*^	< 0.001^*^	0.783	0.721
	Stance T. var (%)	< 0.001^*^	0.031^*^	0.057	0.427	0.087	0.242
	Stance T. med (s)	0.036^*^	< 0.001^*^	0.018^*^	< 0.001^*^	0.409	0.284
	Swing T. me (s)	0.800	0.017^*^	0.009^*^	0.775	0.003^*^	0.014^*^
	Swing T. var (%)	0.050	0.103	0.005^*^	0.614	0.740	0.034^*^
	Swing T. med (s)	0.113	0.002^*^	0.022^*^	0.488	0.094	0.034^*^
	DS T. me (s)	< 0.001^*^	0.018^*^	< 0.001^*^	< 0.001^*^	0.038	0.021^*^
	DS T. var (%)	0.005	0.382	0.153	0.885	< 0.001^*^	0.130
	DS T. med (s)	< 0.001^*^	0.007^*^	< 0.001^*^	< 0.001^*^	< 0.001^*^	0.001^*^
	SS T. me (s)	< 0.001^*^	0.001^*^	0.580	0.023^*^	0.753	0.134
	SS T. var (%)	0.259	0.049^*^	0.263	0.071	0.534	0.815
	SS T. med (s)	0.497	< 0.001^*^	0.740	0.053	0.666	0.102
	Step T. me (s)	< 0.001^*^	0.020*	< 0.001^*^	0.008^*^	0.800	0.597
	Step T. var (%)	0.039^*^	0.281	0.384	0.223	0.674	0.164
	Step T. med (s)	< 0.001^*^	0.281	< 0.001^*^	0.223	0.018^*^	0.164
	Step T. sym me	0.239	0.568	0.032^*^	0.014^*^	0.455	0.015^*^
	Step T. sym var (%)	0.260	0.009^*^	0.049^*^	0.597	0.586	0.102
	Step T. sym med	0.003	0.450	< 0.001^*^	< 0.001^*^	0.088	< 0.001^*^
	Stride T. me (s)	< 0.001^*^	0.024^*^	< 0.001^*^	0.002^*^	0.986	0.307
	Stride T. var (%)	0.443	0.721	0.423	0.172	0.888	0.214
	Stride T. med (s)	< 0.001^*^	0.031^*^	< 0.001^*^	0.016^*^	0.123	0.796
	Stride T.reg me	0.817	0.135	0.302	0.763	0.853	0.283
	Stride T. reg var (%)	0.740	0.855	0.650	0.515	0.853	0.740
	Stride T. reg med	0.607	0.039^*^	0.351	0.556	0.544	0.079
Micro spatial	Step L. me (cm)	< 0.001^*^	0.603	< 0.001^*^	0.004^*^	0.134	0.024^*^
	Step L. var (%)	0.546	0.002^*^	0.720	< 0.001^*^	0.969	0.772
	Step L. med (cm)	< 0.001^*^	0.516	< 0.001^*^	0.048^*^	0.338	0.331
	Step L. sym me	0.343	0.067	0.029^*^	0.217	0.241	0.931
	Step L. sym var (%)	0.862	0.025^*^	0.057	0.219	0.228	0.631
	Step L. sym med	0.041^*^	0.115	0.048^*^	0.213	0.741	0.853
	Step W. me (m)	< 0.001^*^	0.001*	< 0.001^*^	0.001*	0.704	0.663
	Step W. var (%)	< 0.001^*^	0.097	0.011^*^	0.963	0.098	0.356
	Step W. med (m)	< 0.001^*^	0.003^*^	< 0.001^*^	0.024^*^	0.813	0.807
	Step H. me (m)	0.046^*^	0.990	0.063	0.143	0.526	0.356
	Step H. var (%)	0.147	0.002^*^	< 0.001^*^	0.284	0.112	0.115
	Step H. med (m)	0.034^*^	0.363	0.318	0.515	0.265	0.063
	Stride L. me (cm)	< 0.001^*^	0.618	< 0.001^*^	0.002^*^	0.191	0.018^*^
	Stride L. var (%)	0.960	0.107	0.118	0.008^*^	0.203	0.796
	Stride L. med (cm)	< 0.001^*^	0.597	< 0.001^*^	0.001^*^	0.060	0.003^*^
	Stride L. reg me	0.110	0.037^*^	0.028^*^	0.002^*^	0.534	0.440
	Stride L. reg var (%)	0.732	0.508	0.497	0.033^*^	0.391	0.157
	Stride L. reg med	0.029^*^	0.035^*^	0.021^*^	0.001^*^	0.769	0.396
Micro Spatiotemporal	Step V. me (cm/s)	< 0.001^*^	0.017^*^	< 0.001^*^	< 0.001^*^	0.178	0.151
	Step V. var (%)	0.488	0.104	0.137	0.656	0.476	0.263
	Step V. med (cm/s)	< 0.001^*^	0.172	< 0.001^*^	0.001^*^	0.422	0.067
	Stride V. me (cm/s)	< 0.001^*^	0.021^*^	< 0.001^*^	< 0.001^*^	0.382	0.082
	Stride V. var (%)	0.488	0.015^*^	0.007^*^	0.263	0.105	0.438
	Stride V. med (cm/s)	< 0.001^*^	0.136	< 0.001^*^	< 0.001^*^	0.823	0.023^*^

^*^Significant difference at *p*-value < 0.05. HC, healthy control; MCI, mild cognitive impairment; AD, Alzheimer's disease; CI, cognitive impairment; *T*, time; *L*, length; *W*, width; *H*, height; *V*, velocity; me, mean; var, variability; sym, symmetry; reg, regularity; DS, double support; SS, single support.

#### 2.3.2 Selecting significant gait markers

To control for potential confounding effects, ANCOVA was applied to adjust all gait markers for gender, BMI, education, and GDS (39). The Shapiro–Wilk test was used to assess the normality of each gait marker (46), after which appropriate statistical analyses were conducted to identify markers showing significant group differences. For pairwise comparisons (CI vs. HC and AD vs. MCI), independent samples *t*-tests were used for normally distributed markers, and Mann-Whitney U tests were used for non-normal markers (47). For comparisons across the three groups (HC, MCI, AD), one-way ANOVA or Kruskal-Wallis tests were applied depending on the distribution of the data. Categorical gait markers were evaluated using the chi-square test (48). A significance threshold of *p* < 0.05 was used for all tests. Following this step, several significant gait markers were found to be highly correlated (correlation coefficient > 0.95). Since redundancy can negatively impact the performance of machine learning models, only one marker was retained from each group of highly correlated markers. In each case, the marker with the lowest *p*-value was selected. This final non-redundant subset of markers is referred to as the selected significant gait markers and was used in subsequent analyses. We additionally applied Benjamini–Hochberg false-discovery-rate (FDR) correction (49) to the per-marker *p*-values. We emphasize that correlation pruning at |*r*|>0.95 and multiple-comparison correction address different aspects of the problem (redundancy vs. Type-I error rate) and are not interchangeable. Of the 96 markers retained by the two-step selection, 72 survive FDR control at the conventional threshold q ≤ 0.05 and 90 at q ≤ 0.10, the more permissive threshold appropriate to marker-discovery analyses of this kind (50). Because the correction was applied *post hoc* and serves as a sensitivity analysis rather than as the selection criterion, the marker set used for classification and the accuracies reported in Table 3 are unchanged. Per-marker *q*-values at both thresholds are given in Supplementary Section S2.

**Table 3:** Performance of the SVM-based classification framework for distinguishing CI from HC and mild AD from MCI, using ST gait markers (A), DT gait markers (B), their combination (C), and benchmark cognitive screening scores of MoCA (D) and MMSE (E).

Groups	Accuracy	Sensitivity	Specificity	Precision (PPV)	*F*-score	NPV	LR+	LR–
A. Single-task gait markers								
CI vs. HC	79.45	81.39	76.67	83.33	82.35	74.19	3.49	0.243
Mild AD vs. MCI	88.37	84.44	84.00	80.95	87.18	88.23	5.28	0.185
**Overall accuracy = 83.91%**								
B. Dual-task gait markers								
CI vs. HC	84.93	83.72	86.67	90.00	86.75	78.79	6.28	0.188
Mild AD vs. MCI	90.69	88.89	92.00	88.89	88.89	92.00	11.11	0.121
**Overall accuracy = 87.81%**								
C. Combining dual-task and single-tasks gait markers								
CI vs. HC	89.04	88.37	90.00	92.68	90.48	84.37	8.84	0.129
Mild AD vs. MCI	93.02	94.44	93.00	89.47	91.90	95.87	13.49	0.060
**Overall accuracy = 91.03%**								
D. MoCA scores								
CI vs. HC	83.00	76.00	90.00	86.10	80.36	72.35	7.60	0.267
Mild AD vs. MCI	79.50	75.00	84.00	82.60	76.58	82.35	4.69	0.298
**Overall accuracy = 81.25%**								
E. MMSE scores								
CI vs. HC	80.50	66.00	95.00	93.18	76.32	66.09	13.20	0.358
Mild AD vs. MCI	68.50	75.00	62.00	61.04	65.68	77.50	1.97	0.403
**Overall accuracy = 74.50%**								

Reported values represent mean performance across five-fold subject-wise cross-validation.

CI, cognitive impairment; HC, healthy control without cognitive impairment; MCI, mild cognitive impairment; AD, Alzheimer's disease; PPV, positive predictive value; NPV, negative predictive value; LR+, positive likelihood ratio; LR–, negative likelihood ratio.

#### 2.3.3 Machine learning for diagnosis prediction

A sequential machine learning framework was developed to classify participants based on gait markers derived from single- and dual-task curve walking. The classification process consisted of two stages. First, a support vector machine (SVM) model (51) was trained to distinguish individuals with cognitive impairment (CI; MCI or mild AD) from HC. In the second stage, a separate SVM model was used to differentiate between participants with MCI and those with mild AD.

Both models were trained and evaluated using five-fold subject-wise cross-validation. Participants were divided into five non-overlapping folds. In each iteration, four folds were used for training, and the remaining fold was held out for testing. This process was repeated five times to ensure that each participant was included in the test set exactly once. Importantly, marker selection was performed within the training folds only to avoid data leakage and to preserve the integrity of the validation process. The SVM models were optimized using a grid search over hyperparameters (e.g., kernel type and regularization parameter) based on performance on the training data within each fold.

Model performance was reported as the average across all folds, using standard classification metrics: accuracy, sensitivity, specificity, precision (positive predictive value, PPV), negative predictive value (NPV), positive and negative likelihood ratios (LR+, LR–), *F*-score, and area under the receiver operating characteristic curve (AUC). A *post-hoc* power analysis quantifying per-marker Cohen's *d* and achieved statistical power at α = 0.05 for the headline contrasts is reported in Supplementary Section S4. A model-based permutation-importance analysis of the trained classifiers is reported in Supplementary Section S6. The *F*-score, defined as the harmonic mean of precision and sensitivity, provides a balanced measure of a model's ability to correctly identify positive cases while minimizing false positives; higher *F*-scores indicate better classification performance. The AUC reflects the model's ability to discriminate between classes across all decision thresholds, where a value closer to 1.0 indicates superior separability between groups.

To benchmark our approach, we evaluated MoCA and MMSE scores using commonly used clinical thresholds (25–27 for CI detection and 22–24 for AD detection), and computed accuracy, sensitivity, specificity, precision, and *F*-score. Because these screening tools were applied using fixed thresholds, AUC and ROC analyses were not applicable.

## 3 Results

### 3.1 Gait marker differences among diagnostic groups

We analyzed 52 ST and 104 DT gait markers—including DT cost metrics—derived from participants' curve walking data. Supplementary Table S1 provides the mean and standard deviation values of extracted gait markers and their (*p* < 0.05) values for AD vs. MCI vs. HC. Table 2 summarizes statistical comparisons focused on two clinically meaningful stages: (1) identifying cognitive impairment (CI, defined as MCI or AD) vs. HC, and (2) differentiating between AD and MCI. The tables categorize markers by type (macro, micro-temporal, micro-spatial, and micro-spatiotemporal) and highlight significant differences (*p* < 0.05) by an asterisk. Supplementary Table S2 provides *p*-values for all pairwise comparisons (MCI vs. HC, AD vs. HC, AD vs. MCI).

We identified 35 ST and 48 DT markers that were significantly different among HC, MCI, and AD groups; 33 ST and 42 DT for CI vs. HC; and 30 ST and 41 DT for AD vs. MCI Figure 2. A presents the distribution of significant and selected gait markers across various domains for the three group comparisons: (a) AD vs. MCI vs. HC, (b) CI vs. HC, and (c) AD vs. MCI. In each comparison, the total height of each bar represents the number of statistically significant markers, with the stacked darker segments indicating the subset of selected (non-redundant) markers retained after correlation analysis. As illustrated in Figure 2. A, the number of selected markers is 22 ST and 34 DT for the three-class comparison (AD vs. MCI vs. HC), 20 ST/27 DT for CI vs. HC, and 19 ST/30 DT for AD vs. MCI. Notably, a substantial portion of selected features remained within the micro-temporal and micro-spatial categories, underscoring the importance of fine-grained gait dynamics in detecting cognitive impairment.

**Figure 2:**
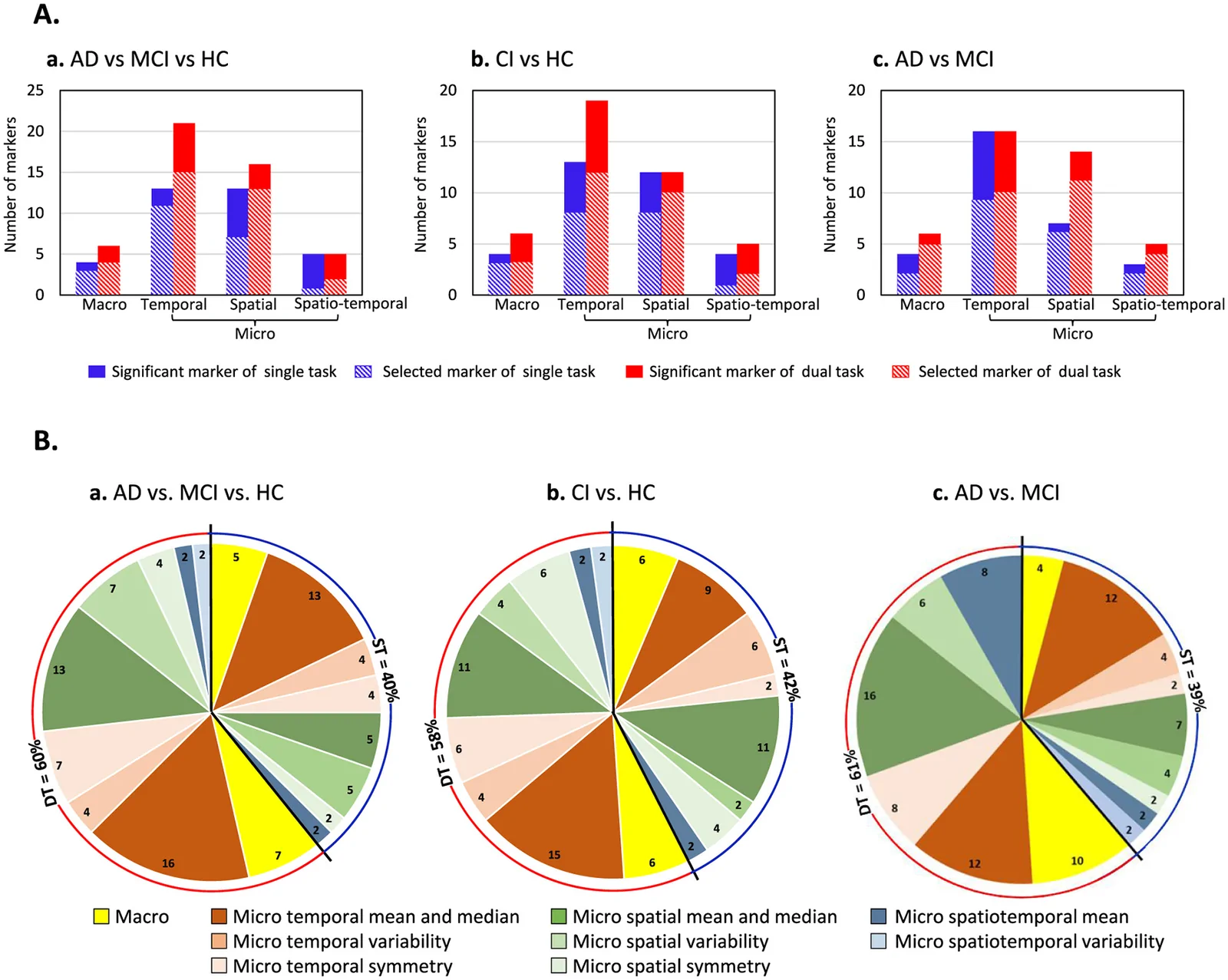
**(A)** Distribution of significant and selected markers from single and dual task curve walking over the marker types. **(B)** The percentage of selected markers for various marker types in single and dual tasking for a. AD. vs. MCI vs. HC, b. CI vs. HC, and c. AD vs. MCI.

To further characterize the selected gait markers, we analyzed their types across mean/median, variability, and symmetry measures. Figure 2B presents the percentage distribution of selected ST and DT markers across these categories. DT markers made up the majority of selected features—60%, 58%, and 61% for AD vs. MCI vs. HC, CI vs. HC, and AD vs. MCI, respectively—highlighting the increased diagnostic value of gait under cognitive load. Most selected markers were related to mean or median values, followed by variability and symmetry. Notably, the share of variability markers increased under DT conditions: from 9 in ST to 13 in DT for AD vs. MCI vs. HC, and from 4 in ST to 5 in DT for CI vs. HC. Symmetry markers also increased with dual-tasking while it changed from 6 in ST to 11 in DT for AD vs. MCI vs. HC, 2 in ST to 5 in DT for CI vs. HC, and 2 in ST to 4 in DT for AD vs. MCI, reflecting growing motor coordination deficits with cognitive decline.

Across macro markers, curve walking duration emerged as the most discriminative ST and DT marker for both CI vs. HC and AD vs. MCI. Within micro-temporal markers, double support time (mean and variability) was most significant for CI vs. HC, whereas stance time (ST) and double support time (DT) were most discriminative for AD vs. MCI. Among micro-spatial markers, step width (ST and DT) was the strongest discriminator for both comparisons. In addition, DT step length was most significant for CI vs. HC, and DT step length variability for AD vs. MCI. In the spatiotemporal domain, step and stride velocity—in both ST and DT—were among the most consistently significant markers across all comparisons.

There were several shared markers. Ten ST and fifteen DT markers were significant in both CI vs. HC and AD vs. MCI comparisons. These included ST and DT curve walking duration (and DT cost), velocity and cadence (ST/DT), DT step number and cost, temporal markers such as ST stance time variability and DT double support time (median, variability, cost), ST and DT step time, stride time, and spatial/spatiotemporal markers such as step width, stride length, and velocity.

We also identified comparison-specific markers. For CI vs. HC, 7 ST and 7 DT markers were uniquely significant, including ST step time variability and symmetry, ST double support time variability, DT swing time, DT step time and double support costs, ST step length and height, ST stride length, ST/DT step width variability, DT step height variability, DT step length symmetry, and DT stride velocity variability. In contrast, AD vs. MCI had 5 ST and 9 DT uniquely significant markers, including ST and DT single support time, ST single support time variability, ST step time symmetry variability, DT step time symmetry cost, DT stance time variability, ST/DT step length variability, DT step length cost, and DT stride length and velocity metrics (variability, regularity, and cost).

Dual-tasking significantly affected multiple gait markers across all groups. Eleven markers showed group-wise DT-related differences for AD vs. MCI vs. HC; seven markers for CI vs. HC; and eleven for AD vs. MCI. These included macro indicators such as curve walking duration and step count, as well as micro-temporal and spatial markers related to step time, stride length, swing time, and double support time.

To illustrate these findings, Figure 3 presents representative examples of the most discriminative gait markers across the three groups. Curve walking duration (Figure 3A) increased progressively from HC to MCI to AD, with DT cost worsening from -2.34 ± 25.99% (*mean*±*STD* for HC) to -18.54 ± 28.30% (MCI) and -55.65 ± 57.73% (AD), reflecting increasingly impaired dual-task performance with greater cognitive impairment. Stance time mean (Figure 3B) demonstrated the strongest micro-temporal difference, increasing under dual-tasking 0.57 → 0.83 s (DTC = −59.06 ± 75.66 for HC), 0.64 → 0.97 s (DTC = −70.77 ± 88.02 for MCI), and 1.09 → 1.69 s (DTC = −81.15 ± 74.84 for AD). For micro-spatial markers, step width (Figure 3C) decreased with increasing impairment (DT: 21 cm → 17 cm → 13 cm). Stride velocity (Figure 3D) showed the most pronounced spatiotemporal differences and DT decline, dropping from 45.52 to 37.17 cm/s in HC (DTC = −17.757 ± 13.01), 35.47 to 28.44 cm/s in MCI (DTC = −16.17 ± 27.15), and 30.41 to 21.27 cm/s in AD (DTC = −28.75 ± 18.79).

**Figure 3:**
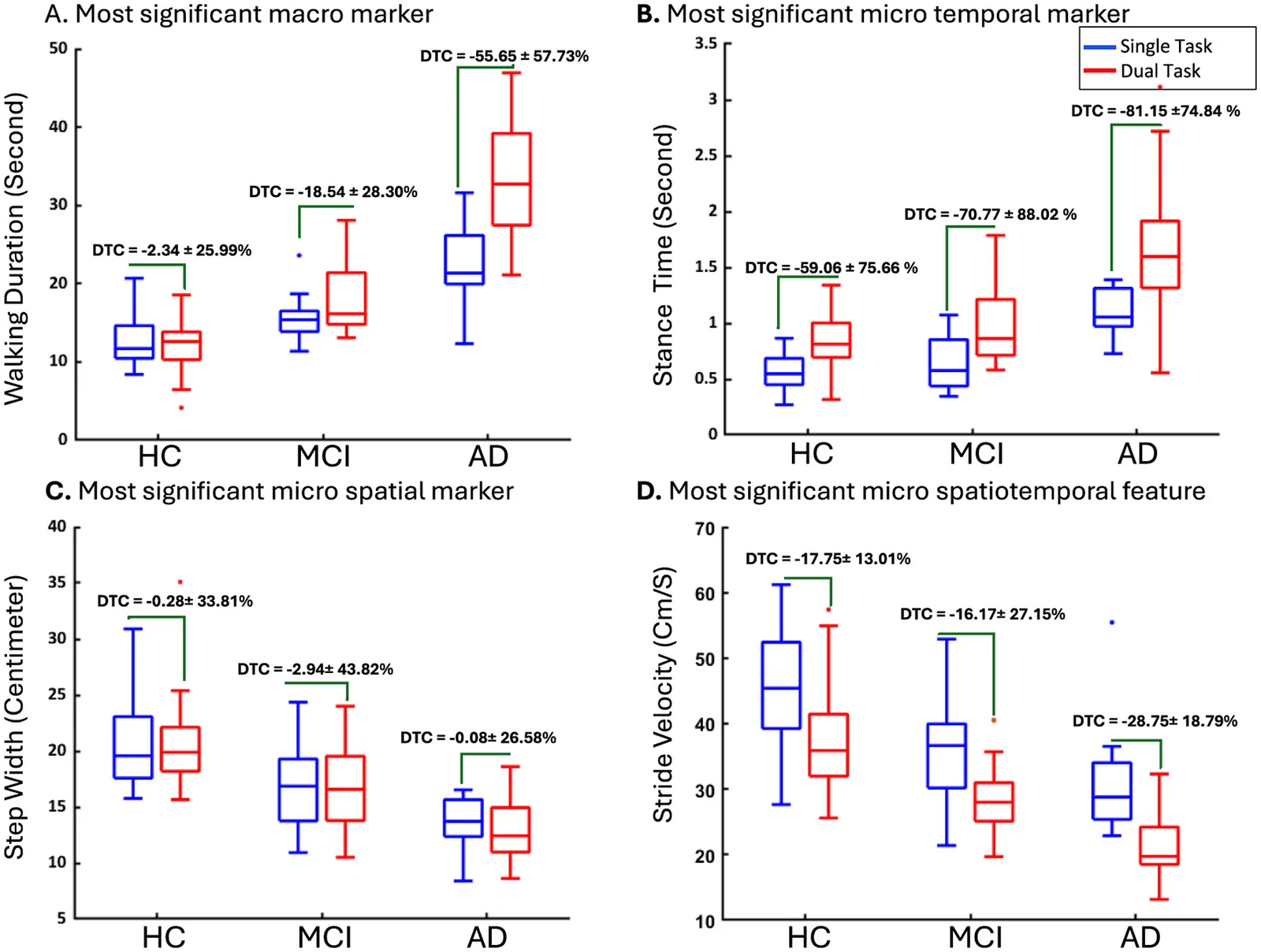
**(A)** The most significant macro, **(B)** micro temporal, **(C)** micro spatial, and **(D)** micro spatiotemporal markers among HC vs. MCI vs. AD. ^*^DTC is showing the dual-task cost.

### 3.2 Diagnostic performance of gait-based machine learning models

Building on the sequential SVM-based classification framework described in the Methods, we evaluated the model's ability to first distinguish cognitively impaired individuals (CI; MCI or mild AD) from HC, and then to differentiate mild AD from MCI. To assess the contribution of DT curve walking, we trained and tested models separately using only ST markers, only DT markers, and then both combined. Table 3 provides a summary of the performance. Supplementary Table S7 provides fold-level variability for each performance metric. Wilson score 95% confidence intervals for each accuracy estimate are reported in Supplementary Table S10; for the combined ST+DT models these are 79.8%–94.3% for CI vs. HC and 81.4%–97.6% for mild AD vs. MCI. As summarized in Table 3, the model trained on ST markers achieved a baseline overall accuracy of 83.91%. Incorporating DT markers improved this to 87.81%, and combining both ST and DT markers yielded the best overall performance at 91.03%.

For CI vs. HC classification, combining ST and DT markers resulted in an accuracy of 89.04% and sensitivity of 88.37%, compared to 84.93% accuracy and 83.72% sensitivity using DT markers alone. The model's performance in distinguishing mild AD from MCI followed a similar trend. DT-based models achieved 90.69% accuracy and 88.89% sensitivity, while combining both marker sets improved accuracy to 93.02% and sensitivity to 94.44%.

Figure 4 presents the ROC curves for each model setup. For CI vs. HC, the AUC increased from 0.88 (ST) to 0.94 (DT) and reached 0.96 when combining ST and DT. For mild AD vs. MCI, AUC values were similarly high: 0.92 (ST), 0.94 (DT), and 0.95 (combined), confirming that dual-tasking adds diagnostic value, and that combining gait markers under both conditions leads to the strongest discriminative power.

**Figure 4:**
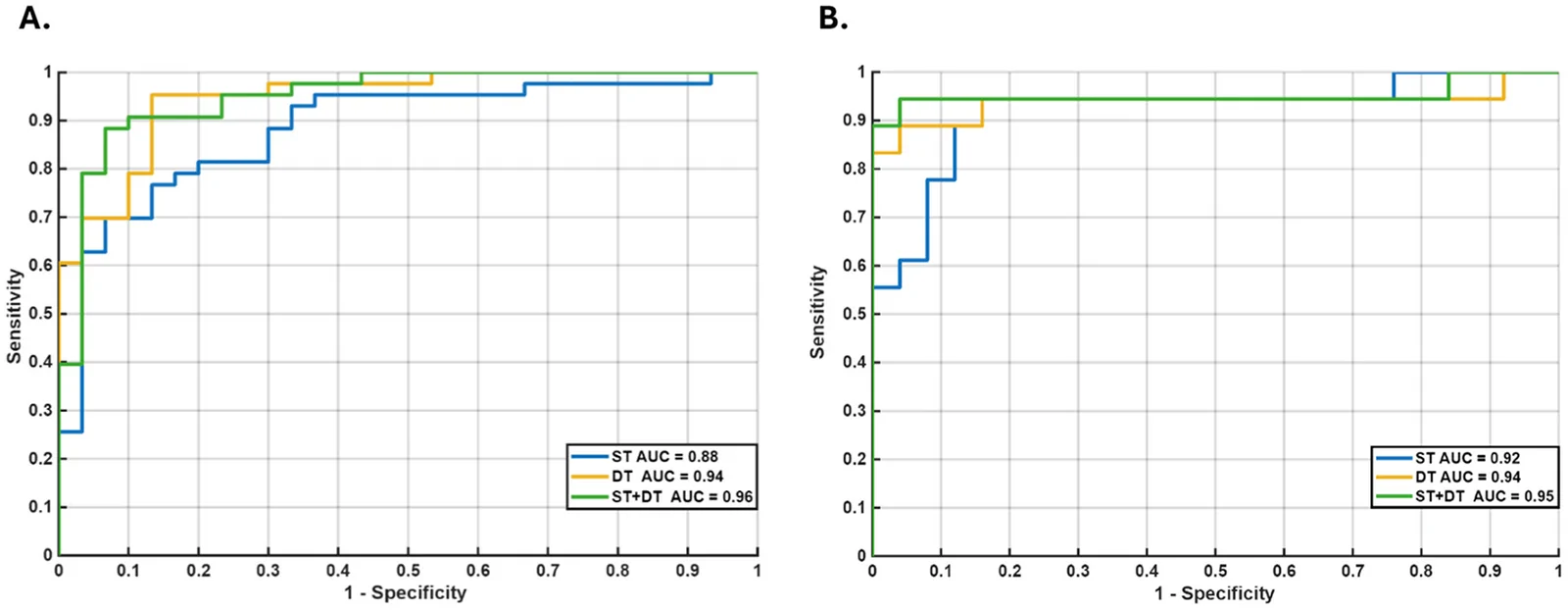
ROC analysis for CI vs. HC **(A)** and mild AD vs. MCI **(B)** and their area under curve values (AUC).

To benchmark against established cognitive screening tools, we evaluated classification performance using MoCA and MMSE scores (Tables 3C, D). MoCA achieved 81.25% overall accuracy and 76.00% sensitivity for CI detection, while MMSE yielded 74.50% accuracy and 66.00% sensitivity. Both tools showed 75.00% sensitivity in distinguishing mild AD from MCI. These results indicate that gait-based models—especially those incorporating DT or combined ST/DT markers—outperform MoCA and MMSE in both classification accuracy and sensitivity. The improvement in sensitivity (e.g., 94.44% vs. 75.00%) is particularly important for early identification of individuals at risk for progression.

## 4 Discussion

Early and accurate detection of cognitive impairment, including mild cognitive impairment and Alzheimer's disease, remains a critical yet unmet need in clinical practice. Despite its importance, cognitive screening is often underutilized: over 50% of primary care physicians do not perform routine screening unless prompted by overt symptoms or complaints from patients or family members (33, 37). Confirmatory diagnostic tools such as MRI and PET scans are costly, time-consuming, and often inaccessible in many clinical settings (15, 16), further delaying timely intervention.

To address these limitations, we proposed and evaluated a novel, non-invasive assessment method based on DT curve walking captured by a single depth camera and analyzed using machine learning. This study builds on our prior work demonstrating that curve walking is more discriminative than straight-path walking for identifying early cognitive impairment (38). Here, we extend that finding by providing a comprehensive analysis of gait during ST and DT curve walking across HC, individuals with MCI, and those with AD. To our knowledge, this is the first study to apply DT curve walking on an oval path using a depth camera to distinguish between HC, individuals with MCI, and those with mild AD. We applied our study design to a cohort of 73 older adults spanning the full spectrum of cognitive status (HC, MCI, and mild AD). Gait data collected under ST and DT conditions were processed using custom software to extract a rich set of macro and micro gait markers. These markers were then used to train machine learning models capable of predicting CI status and its substage without relying on clinical history or subjective assessments. The following sections summarize the key findings from this study and discuss their implications for early detection of cognitive impairment.

### 4.1 Enabling scalable and detailed gait assessment using a single-camera system

This study demonstrates that a single Kinect v.2 depth camera can reliably support detailed gait assessment in cognitively impaired and cognitively intact older adults during curved-path walking. The system enabled full-body, joint-level motion capture without the need for wearable devices or specialized setup, allowing for the extraction of a comprehensive set of gait markers—including macro, micro-temporal, micro-spatial, and spatiotemporal markers. Compared to prior studies that relied on straight-path walking and pressure-sensitive mats or body-worn sensors (52, 53), our approach offers broader coverage of gait dynamics while preserving natural movement. The ability to analyze turning gait under both single- and dual-task conditions with minimal hardware setup allowed us to identify subtle motor-cognitive deficits associated with MCI and mild AD. The system is therefore suited to detailed gait assessment in supervised settings such as primary care and memory-clinic offices; extension to less-controlled environments would require the additional validation outlined in Section 4.6.

Because the depth camera is the sole measurement instrument used here, its accuracy requires consideration. Kinect-based motion capture provides a low-cost, markerless alternative to conventional marker-based systems, but it is less accurate than laboratory-grade optical systems, particularly during dynamic movement. This limitation has been quantified in several validation studies. Galna et al. and Gonzalez-Jorge et al. reported that Kinect v.2 achieves good spatial and temporal accuracy for static postures, but that accuracy declines during dynamic activities such as walking and that spatial error grows with subject-to-camera distance (54–56). Otte et al. and Capecci et al. found Kinect v.2 acceptable for clinical motor-function assessment, although accuracy is reduced by self-occlusion and off-axis movement (57, 58), and Dolatabadi et al. and Usami et al. reported good concurrent validity with marker-based systems for spatiotemporal gait parameters specifically (59, 60). In our own recordings, spatial error reached approximately 25 mm at the greatest walking distance from the sensor, and the position of the camera relative to the oval path was chosen with this in mind. Our recent validation study likewise found the Kinect camera comparable in accuracy and reliability to gait-measurement systems in routine use in research clinics, including wearable sensors and instrumented walkways (53).

Whether this level of accuracy is adequate depends on the size of the differences to be resolved. Gait alterations associated with MCI and early cognitive decline, including reduced gait speed, shortened stride length, increased step-time variability, and reduced gait symmetry and regularity, are reported to differ from healthy-control values by margins on the order of several centimeters or tens of milliseconds. Such margins exceed the spatial error reported for Kinect v.2 under favorable conditions (1–3 cm) and also exceed the worst-case error in our own setup (≈25 mm). The differences we observed are consistent with this: the discriminative markers reported in Section 3.1 separate the groups by margins well above the error floor of the sensor, for example a dual-task stride velocity difference of approximately 16 cm/s between HC and mild AD. Measurement error of this kind adds noise to the input markers and therefore attenuates rather than inflates group separation. We consider Kinect v.2 adequate for the group-level contrasts examined here, in line with the Kinect-based dementia-detection literature cited elsewhere in this manuscript (61).

### 4.2 Curve walking enables sensitive detection of cognitive impairment

In this study for the first time, curve walking around an oval path in DT and ST was examined comprehensively including macro, micro temporal and spatial, and micro spatiotemporal markers for three study groups of HC, MCI and mild AD. In comparison, previous studies focused on straight walking, and a smaller collection of markers had been examined (26, 28, 29). They mostly focused on markers like average velocity, cadence, step or stride time and length, and the symmetry of stride time or length. In comparison, we extracted not only mean of step and stride, their median and variability were also calculated. In addition, various parameters of gait cycles like single and double support, stance and swing phases were studied in more detail for their mean, median and variability. For symmetry and regularity, we examined the mean, median and variability of both micro temporal and micro spatial marker while previous studies only focused on mean of micro temporal markers of stride or step (27, 62).

Our finding showed that even majority of ST markers were significantly different in curve walking in comparison to previous studies findings which no significant differences were observed for most of the markers in ST straight walking between HC and CI (26, 63). These results can be because of the more sensitivity of curve walking for detection the decrease in cognitive ability among older adults which were also mentioned by Lee et al. (37) and proofed in our recent study too (38). These differences between sensitivity of curve walking and straight walking also have been observed in transition state like turning during performing the TUG test between CI and HC in studies like Mirelman et al. and Ansai et al. while no significant differences were seen in markers of straight walking subphases of TUG in ST condition between CI and HC groups (48, 64). These differences between curve and straight walking may be because curve walking is not rhythmic and in a repeated pattern like straight walking, and the curve walking involve multiple cognitive skills simultaneously like finding the direction and control balance while the participant continue walking (5, 37).

### 4.3 Single-task curve walking reveals key gait differences in cognitive impairment

Our findings from ST curve walking reveal consistent and meaningful gait differences between older adults with CI and HC. These differences are evident across multiple gait domains and reinforce prior reports while also extending them through the use of a curved walking path, which increases cognitive and postural demands compared to traditional straight walking. Consistent with earlier studies on straight-path gait, we found that individuals with CI required more time and took more steps to complete the walking task, accompanied by significantly reduced velocity and cadence (25, 65–68). These results align with those reported by Choi et al. and Kim et al., who also identified prolonged gait duration and step count as reliable indicators of CI (65, 66).

In addition to macro gait markers, our analysis of micro-temporal markers during curve walking showed longer stance and swing phases in the CI group, mirroring findings by Lindh-Rengifo et al. (67) and Beauchet et al. (68). Micro-spatial markers—including step and stride lengths—were also significantly shorter in CI participants compared to HCs. However, as reported in prior studies, no major differences in these spatial metrics were observed between MCI and AD groups, suggesting a possible early plateau in spatial gait decline (25, 65).

Importantly, our study adds to the limited literature on curved-path walking in cognitive impairment. While most prior research has focused on straight walking, only a few studies have examined turning segments during gait. For instance, Wang et al. found reduced turning velocity in individuals with CI, which is in agreement with our observation of slower gait in curved-path walking (30). Unlike linear walking, curve walking imposes continuous demands on balance, motor coordination, and spatial navigation—functions known to deteriorate in CI but often not stressed during straight-path protocols.

In contrast to our results, Gillain et al. did not find significant group differences in gait velocity or stride length during straight-path ST walking between MCI and HC groups, although they did observe differences between MCI and AD participants (62). This discrepancy further highlights the enhanced sensitivity of curved-path walking. The rhythmic and repetitive nature of straight walking may mask early cognitive deficits that become evident under the more dynamic and cognitively taxing conditions of curve walking. Our recent study directly comparing straight and curve walking under ST conditions confirmed this interpretation, showing better group separation using curved-path markers (38).

Finally, we found that the durations of nearly all subphases of the gait cycle—excluding swing and single-support phases—were significantly longer in CI participants, especially in the comparison of HC vs. CI and AD vs. MCI. These alterations in gait dynamics extended to spatiotemporal markers, where both step and stride velocities were reduced among individuals with cognitive impairment, corroborating earlier reports by Lindh-Rengifo et al. (67). In summary, our results validate previously established gait changes associated with CI but demonstrate that curved-path walking, even under single-task conditions, elicits more pronounced differences between clinical groups. These findings underscore the utility of curve walking as a more sensitive functional assessment for early detection of cognitive decline.

### 4.4 Impact of dual-tasking on gait variability in curve walking

DT influenced the variability of micro-level gait markers in a contrast-dependent manner. For CI vs. HC, the number of selected variability markers was modestly higher under DT than ST, indicating that dual-task curved walking surfaces additional variability-based discriminators between cognitively impaired individuals and healthy controls. For the AD vs. MCI contrast, the number of selected variability markers was comparable between conditions, but the identity of the markers shifted—temporal-variability markers prominent in ST were partially replaced by spatial- and regularity-variability markers in DT, suggesting a re-distribution rather than an amplification of variability-based group differences. As illustrated in Figure 2B, the number of selected significant variability markers increased from 9 in ST to 13 in DT when comparing HC, MCI, and AD. A similar pattern emerged for CI vs. HC, where DT resulted in 5 significant variability markers vs. 4 in ST. For CI vs. HC, this pattern indicates that DT walking surfaces additional variability-based discriminators that are not evident during ST walking. The selected variability markers under DT for CI vs. HC include swing-time variability, step-width variability, step-height variability, and stride-velocity variability, with swing-time and double-support variability emerging under DT as discriminators that were not selected in ST. The corresponding ST set includes stance-time, double-support, step-time, and step-width variability. The shift in the variability marker set between ST and DT—rather than a simple uniform increase across all variability domains—suggests that cognitive load reorganizes which aspects of gait become most sensitive to group status.

Several additional markers emerged as significant only during DT, including the variability of swing time, step time symmetry, and stride velocity. These markers further differentiated CI from HC participants and were not significant in the ST condition, reinforcing the value of dual-task assessments in uncovering subtle motor-cognitive dysfunction. However, not all markers responded equally to DT. Variability in certain micro-temporal (e.g., single support time, stride time, stride time regularity), micro-spatial (e.g., step and stride length, stride length regularity), and micro-spatiotemporal markers (e.g., step velocity) did not show statistically significant differences between groups, although slight changes were observed after cognitive task introduction. This suggests that while some gait features are highly sensitive to cognitive load, others may remain relatively stable across conditions.

Furthermore, a graded increase in variability was observed with worsening cognitive status. Individuals with AD showed greater variability in several micro-temporal features—including stance time, swing time, single support time, step time, and stride time—compared to those with MCI. For micro-spatial markers, however, only the variability of step length symmetry significantly distinguished AD from MCI, indicating that temporal variability may be a more robust indicator of cognitive decline progression than spatial inconsistencies. These findings are consistent with prior reports by Muir et al., Montero-Odasso et al., and König et al., who noted that gait variability increases more in CI than HC under dual-task conditions (26, 63, 69). However, most previous studies evaluated variability in a limited set of markers and typically relied on straight-path walking. In contrast, our study applied a more comprehensive gait analysis on a cognitively demanding curved path, enabling deeper characterization of variability changes and their relationship to cognitive status. These results underscore the diagnostic potential of detailed variability analysis in curved-path walking for early detection and monitoring of cognitive decline.

In addition to the variability findings above, DT curve walking also revealed a roughly two-fold increase in the number of selected gait-symmetry markers in both CI vs. HC and AD vs. MCI contrasts. Compared to ST walking, the number of selected significant symmetry markers approximately doubled under DT conditions across all diagnostic comparisons (e.g., 6 to 11 markers for AD vs. MCI vs. HC; 2 to 5 for CI vs. HC; and 2 to 4 for AD vs. MCI; see Figure 2B). Specifically, Step Time symmetry Mean and Variability and Step Length symmetry Mean were not selected in ST but became discriminative in DT, while Step Time symmetry Median and Step Length symmetry Median were already selected in ST and remained so under DT. This pattern indicates that DT primarily recruits additional symmetry sub-markers (mean and variability) rather than replacing the ST symmetry findings. Our findings align with those of Gillain et al. (62), who reported significant step time asymmetries under DT for CI vs. HC and AD vs. MCI, but no differences under ST. Similarly, Maquet et al. (27) observed reduced stride time regularity in both ST and DT, with greater asymmetry among individuals with CI. These findings collectively suggest that the added cognitive load of DT tasks disproportionately disrupts bilateral coordination in individuals with MCI or AD.

### 4.5 Dual-task curve walking markers enable accurate, objective group separation via machine learning

While the core aim of this study was to explore whether curved-path walking—particularly under DT conditions—could reveal clinically relevant gait markers of cognitive impairment, machine learning served as a powerful validation tool to test the discriminative strength of these markers. Using SVM models, we assessed whether macro- and micro-level gait markers, derived from both ST and DT curve walking, could accurately classify older adults into diagnostic groups. Our results confirmed that within the curved-walking paradigm, dual-task conditions reveal richer and more distinctive gait information about cognitive status than single-task conditions alone. The present study does not directly compare curved DT walking with straight DT walking, as straight-DT data were not collected. The superiority of curved over straight walking was established under single-task conditions in our prior work (38), and the added value of dual-tasking is demonstrated here within the curved-walking context. A definitive claim that curved DT walking outperforms straight DT walking must await a future study incorporating both conditions. Models trained on DT markers outperformed those based on ST markers in both stages of classification. For instance, the accuracy for detecting CI vs. HC improved from 79.45% using ST markers to 84.93% with DT markers. Similarly, discrimination between AD and MCI improved from 88.37% (ST) to 90.69% (DT). When combining both ST and DT markers, performance further improved, achieving 89.04% accuracy for CI vs. HC and 93.02% for AD vs. MCI, with sensitivities of 88.37% and 94.44%, respectively. These results confirm that the added cognitive and balance demands of curved dual-task walking reveal subtle motor-cognitive changes that are otherwise difficult to detect—especially in early-stage impairment. The machine learning models were not intended as black-box classifiers but as an objective lens through which the diagnostic value of the gait markers could be evaluated.

Importantly, the models outperformed conventional tools such as MoCA and MMSE in sensitivity and *F*-score, emphasizing the limitations of static cognitive screens in capturing dynamic, real-world deficits. The results highlight the promise of curved-path dual-task walking as a non-invasive, accessible, and interpretable screening approach—especially when combined with automated machine learning tools for decision support.

### 4.6 Clinical application and study limitation

The proposed assessment tool—based on Kinect v.2, curved-path walking, and machine learning–offers a promising, non-invasive approach for detecting CI and distinguishing between MCI and mild AD. Its ability to extract comprehensive gait markers using a single depth camera makes it a promising candidate for future deployment in supervised clinical and community environments (e.g., memory clinics and geriatric offices). Direct deployment to less-controlled environments such as assisted-living facilities or older adults' homes would require additional validation under variable lighting, camera-placement, and body-joint-occlusion conditions, and is identified as future work. Compared to traditional tools, this system is low-cost, space-efficient, easy to deploy, and respects user privacy by using joint-level skeletal signals rather than RGB video. As such, it can serve as a valuable prescreening or complementary tool, flagging individuals for more detailed evaluation using conventional diagnostic methods.

Despite its promise, several aspects of this study merit acknowledgment as natural opportunities for the next stage of validation. First, the cohort is subtype-homogeneous: all 25 MCI participants were clinically diagnosed as MCI due to Alzheimer's disease, which strengthens internal validity for the present analyses but warrants future testing in non-amnestic MCI cohorts to establish generalization across MCI subtypes (11). Second, although a *post-hoc* power analysis confirms large effect sizes for both headline contrasts (mean Cohen's *d* of 1.16 for CI vs. HC and 1.01 for AD vs. MCI, with achieved power at α = 0.05 of 0.94 and 0.72, respectively; see Supplementary Section S4), the modest AD subgroup (*n* = 18) motivates external validation in a larger multi-site cohort, which we are now actively initiating. Third, all gait recordings were obtained under controlled conditions with a fixed camera placement and an oval walking path; deployment to less-controlled environments—including variable lighting, camera placement, body-joint occlusion, and free-form walking trajectories—is the next translational step. Fourth, all 73 participants spontaneously elected to walk clockwise, which removes direction as a within-cohort confound but reserves validation on counter-clockwise gait for follow-up work. Fifth, the single-classifier (SVM) design was chosen intentionally to demonstrate that a simple, interpretable model can achieve clinically meaningful performance on curved dual-task gait markers; comprehensive benchmarking against alternative classifiers is a complementary direction for methodological follow-up work. Finally, this is a cross-sectional study, and a direct curved-DT-vs-straight-DT comparison was not performed; a planned 2 × 2 factorial (path type × task condition) and longitudinal follow-up of this cohort represent the natural extensions of the present work and would complete the evidence base for adopting curved dual-task walking as a definitive cognitive-screening paradigm.

The spatial resolution of the depth camera also bounds what can be measured. Although Kinect v.2 was sufficiently accurate for the cognition-related gait alterations examined in this study, its resolution (1–3 cm under favorable conditions, and up to approximately 25 mm at greater sensor-to-subject distances in our setup) is not adequate for finer-grained biomechanical analyses requiring sub-centimeter precision. Marker-level differences at or below that scale should therefore be interpreted with caution, and pairing Kinect-based assessment with marker-based or wearable-sensor systems would permit cross-validation at higher resolution.

## 5 Conclusion

This study presents a novel, non-invasive approach for early detection and staging of cognitive impairment in older adults by analyzing single- and dual-task curved-path walking using a single depth camera. In a cohort of 73 participants—including healthy controls, individuals with MCI, and those with mild AD—we demonstrated that curved walking, particularly under cognitively demanding dual-task conditions, exposes subtle gait disturbances that are often missed by conventional straight-path assessments. Unlike rhythmic straight walking, curved walking imposes continuous demands on balance, coordination, and spatial planning, making it more sensitive to early motor-cognitive changes associated with MCI and AD. Our detailed analysis uncovered significant group differences across macro (e.g., gait speed, cadence, and step count) and micro-level markers (e.g., swing phase duration, step length, and stride velocity). Moreover, dual-task curve walking revealed additional discriminative gait markers—particularly in step time symmetry (mean and variability) and step length symmetry (mean)—that were not selected under single-task walking, alongside a contrast-dependent shift in which variability markers were retained, underscoring the diagnostic relevance of the curved dual-task paradigm. By applying machine learning to selected markers, we achieved high classification performance, with accuracies of 89.04% for distinguishing cognitively impaired individuals from healthy controls and 93.02% for separating mild AD from MCI. Together, these findings support curved-path dual-task gait assessment as a promising research approach for early ADRD detection that warrants further validation under less-controlled environmental conditions. Future research should validate this approach in larger and more diverse populations and explore additional walking paradigms to further enhance the sensitivity and specificity of motor-cognitive assessments in aging populations.

## Data Availability

The raw data supporting the conclusions of this article will be made available by the authors, without undue reservation.
