# Supplementary material for: Curve walking gait analysis using depth camera and machine learning for detecting cognitive impairment
Source: Front Neurol. 2026 Sep 29;17:1777635. doi: 10.3389/fneur.2026.1777635 (PMC13658113; doi:10.3389/fneur.2026.1777635)
Supplement: Supplementary file 1 [file Data_Sheet_1.pdf]

Supplementary Material

S1 COMPARISON OF EXTRACTED GAIT MARKERS OF THREE STUDY GROUPS (AD VS. MCI VS. AD) AND BETWEEN ONE GROUP VS. ONE GROUP VIA *P*-VALUES FOR SINGLE AND DUAL-TASK CURVE WALKING

Table S1 showed the mean and standard deviation of the extracted gait markers form curve path walking and their p-values. Also, Table S2 shows the results of comparison of the post-hoc analysis between one group vs. one groups to find the probable significant markers based on the reported p-values.

S2 FDR AUDIT OF SELECTED GAIT MARKERS

As a sensitivity analysis in response to the reviewer's comment about multiple-comparison correction, we applied the Benjamini–Hochberg (BH) false-discovery-rate procedure [B1] across all gait markers within each (contrast, condition) family. Tables S3–S6 list, for each of the four (contrast $\times$ condition) families, all gait markers retained after the two-step (significance + correlation pruning at $|r| > 0.95$) selection procedure. Adjusted q-values are reported for every selected marker at two thresholds. Of the 96 markers retained by the two-step procedure, 72 survive BH-FDR control at $q \leq 0.05$ and 90 at $q \leq 0.10$. We report the more permissive threshold alongside the conventional one because this analysis serves marker discovery rather than confirmatory testing: q values in the range 0.10–0.20 are recommended for exploratory work of this kind [B2], BH control is conservative when tests are positively correlated, as gait markers derived from shared limb dynamics necessarily are, and the modest AD subgroup ($n = 18$) places several markers with large effect sizes in the 0.05–0.10 band through sampling variance alone. The FDR correction was applied post hoc, after the analyses reported in the main article, and functions here as a sensitivity analysis: the marker set passed to the classifiers is defined by the two-step procedure described in Section 2.3.2 of the main article and not by the FDR threshold, so the classification results in Table 3 of the main article are unchanged. The six markers that do not reach $q \leq 0.10$ are

Table S1. Comparison of extracted gait markers from curve walking between three study groups in single and dual task conditions.

Feature		Single-task curve walking				Dual-tasking curve walking				Dual-tasking cost			
**Type**	**Name**	**HC**	**MCI**	**AD**	***p*-value**	**HC**	**MCI**	**AD**	***p*-value**	**HC**	**MCI**	**AD**	***p*-value**
**Macro**	Duration (s)	12.57 ± 3.13	15.48 ± 2.47	22.69 ± 4.97	<0.001*	12.05 ± 3.14	18.13 ± 4.32	33.52 ± 7.30	<0.001*	-2.34 ± 25.99	-18.54 ± 28.3	-55.65 ± 57.73	<0.001*
	Velocity (cm/s)	45.89 ± 8.88	39.27 ± 10.09	33.13 ± 7.96	<0.001*	35.79 ± 7.90	30.14 ± 7.29	23.27 ± 4.27	<0.001*	20.29 ± 17.41	20.84 ± 18.28	26.71 ± 20.52	0.202
	Step number	13.30 ± 2.85	13.97 ± 2.71	18.46 ± 3.71	<0.001*	10.27 ± 2.4	12.66 ± 2.56	20.8 ± 3.41	<0.001*	21.39 ± 16.12	7.62 ± 19.46	-15.05 ± 21.4	<0.001*
	Cadence	62.02 ± 9.73	54.99 ± 11.67	49.70 ± 8.34	<0.001*	49.36 ± 8.68	43.29 ± 9.16	38.01 ± 6.59	<0.001*	19.58 ± 12.34	19.64 ± 15.13	21.51 ± 17.94	0.894
**Micro Temporal**	Stance T. me (s)	0.57 ± 0.16	0.64 ± 0.22	1.09 ± 0.21	<0.001*	0.83 ± 0.24	0.97 ± 0.32	1.66 ± 0.57	<0.001*	-59.06 ± 75.66	-70.77 ± 88.02	-61.15 ± 74.84	0.927
	Stance T. var (%)	49.90 ± 18.75	73.08 ± 20.26	60.59 ± 14.36	<0.001*	62.17 ± 21.55	74.06 ± 22.47	68.85 ± 18.23	0.123	-44.37 ± 92.41	-9.84 ± 48.83	-17.75 ± 36.58	0.136
	Stance T. med (s)	0.53 ± 0.17	0.52 ± 0.23	0.98 ± 0.23	<0.001*	0.75 ± 0.27	0.81 ± 0.36	1.36 ± 0.52	<0.001*	-50.07 ± 75.66	-77.13 ± 98.92	-44.76 ± 64.51	0.428
	Swing T. me (s)	0.54 ± 0.04	0.52 ± 0.07	0.60 ± 0.11	0.038*	0.56 ± 0.07	0.5 ± 0.08	0.51 ± 0.10	0.032*	-2.95 ± 16.7	3.44 ± 15.09	14.41 ± 11.89	0.001*
	Swing T. var (%)	26.09 ± 7.99	32.89 ± 11.12	27.98 ± 6.65	0.052	25.91 ± 9.38	31.73 ± 9.62	33.06 ± 9.58	0.033*	-8.25 ± 49.29	-7.23 ± 46.11	-25.23 ± 53.48	0.492
	Swing T. med (s)	0.51 ± 0.05	0.48 ± 0.06	0.57 ± 0.14	0.001*	0.54 ± 0.07	0.48 ± 0.08	0.50 ± 0.11	0.057	-5.18 ± 18.68	-1.53 ± 15.67	10.13 ± 19.10	0.020*
	DS T. me (s)	0.21 ± 0.04	0.31 ± 0.09	0.37 ± 0.08	<0.001*	0.28 ± 0.09	0.43 ± 0.12	0.76 ± 0.29	<0.001*	-37.75 ± 62.86	-48.74 ± 52.97	-127.96 ± 163.48	0.009*
	DS T. var (%)	80.16 ± 22.57	91.91 ± 21.54	103.46 ± 27.85	0.010*	106.52 ± 28.45	98.68 ± 26.11	97.44 ± 29.16	0.450	-39.43 ± 43.9	-11.06 ± 32.07	-1.32 ± 46.2	0.002*
	DS T. med (s)	0.17 ± 0.05	0.23 ± 0.05	0.29 ± 0.09	<0.001*	0.17 ± 0.10	0.30 ± 0.09	0.63 ± 0.26	<0.001*	-4.75 ± 61.53	-38.75 ± 51.49	-183.02 ± 319	<0.001*
	SS T. me (s)	0.40 ± 0.08	0.35 ± 0.09	0.47 ± 0.14	0.002*	0.42 ± 0.09	0.38 ± 0.09	0.45 ± 0.10	0.042*	-7.89 ± 24.76	-10.68 ± 23.02	0.32 ± 23.63	0.320
	SS T. var (%)	48.99 ± 13.07	48.56 ± 11.29	41.92 ± 9.62	0.102	51.46 ± 12.70	50.72 ± 9.05	45.59 ± 8.83	0.164	-14.2 ± 50.98	-9.13 ± 26.92	-17.87 ± 56.81	0.808
	SS T. med (s)	0.36 ± 0.09	0.34 ± 0.10	0.47 ± 0.16	<0.001*	0.38 ± 0.10	0.37 ± 0.10	0.43 ± 0.12	0.139	-8.7 ± 25.33	-11.94 ± 28.15	2.56 ± 28	0.207
	Step T. me (s)	0.88 ± 0.13	1.05 ± 0.27	1.14 ± 0.16	<0.001*	1.13 ± 0.19	1.34 ± 0.29	1.55 ± 0.26	<0.001*	-28.69 ± 20.4	-31.32 ± 32.19	-41.35 ± 50.92	0.841
	Step T. var (%)	34.08 ± 17.07	40.51 ±10.49	36.61 ± 12.89	0.064	41.30 ± 12.71	45.62 ± 21.88	46.88 ± 11.45	0.326	-57.16 ± 116.28	-19.32 ± 62.5	-45.22 ± 63.52	0.310
	Step T. med (s)	0.83 ± 0.09	0.94 ± 0.20	1.06 ± 0.15	<0.001*	0.98 ± 0.16	1.20 ± 0.22	1.45 ± 0.19	<0.001*	-19.22 ± 18.18	-30.56 ± 29.44	-39.9 ± 35.15	0.042*
	Step T. sym me	0.83 ± 0.03	0.84 ± 0.03	0.84 ± 0.03	0.429	0.82 ± 0.02	0.82 ± 0.05	0.85 ± 0.04	0.004*	1.5 ± 4.15	2.44 ± 6.62	-2.14 ± 4.59	0.017*
	Step T. sym var (%)	14.64 ± 5.93	14.45 ± 5.27	18.49 ± 4.45	0.020*	12.68 ± 4.01	15.56 ± 6.85	16.52 ± 7.13	0.120	-1.26 ± 54.14	-18.52 ± 56.85	-0.99 ± 81.92	0.209
	Step T. sym med	0.87 ± 0.03	0.88 ± 0.03	0.89 ± 0.03	0.008*	0.83 ± 0.03	0.85 ± 0.04	0.9 ± 0.03	<0.001*	3.89 ± 3.42	4.18 ± 4.74	-0.79 ± 3.53	<0.001*
	Stride T. me (s)	1.77 ± 0.26	2.08 ± 0.53	2.28 ± 0.33	<0.001*	2.24 ± 0.38	2.58 ± 0.6	3.08 ± 0.52	<0.001*	-27.43 ± 20.09	-27.93 ± 32.67	-41.38 ± 51.14	0.575
	Stride T. var (%)	26.12 ± 12.36	29.41 ± 10.98	26.64 ± 11.22	0.582	28.07 ± 9.79	30.45 ± 17.77	33.37 ± 9.22	0.215	-54± 165	-18.13 ± 72.61	-59 ± 101	0.447
	Stride T. med (s)	1.67 ± 0.18	1.92 ± 0.42	2.15 ± 0.33	<0.001*	2.08 ± 0.32	2.51 ± 0.46	2.85 ± 0.39	<0.001*	-25.49 ± 20.42	-34.89 ± 34.18	-37.41 ± 39.7	0.289
	Stride T.reg me	0.76 ± 0.09	0.74 ± 0.09	0.78 ± 0.06	0.357	0.69 ± 0.12	0.72 ± 0.13	0.71 ± 0.09	0.564	8.77 ± 16.67	1.15 ± 21.77	7.74 ± 15.98	0.284
	Stride T. reg var (%)	21.75 ± 14.39	18.65 ± 9.16	19.19 ± 9.93	0.933	20.04 ± 10.89	24.28 ± 17.78	21.28 ± 9.04	0.900	20.55 ± 30.85	0.43 ± 276.7	6.37 ± 27.63	0.952
	Stride T. reg med	0.75 ± 0.11	0.74 ± 0.11	0.80 ± 0.05	0.139	0.70 ± 0.14	0.74 ± 0.14	0.72±0.11	0.554	5.91 ± 18.8	-1.90 ± 23.73	9.71 ± 15.81	0.149
**Micro Spatial**	Step L. me (cm)	37.58 ± 4.97	32.91 ± 3.46	32.27 ± 4.51	<0.001*	36.93 ± 4.34	31.92 ± 3.36	28.65 ± 3.5	<0.001*	0.46 ± 14.93	2.25 ± 12.64	10.56 ± 9.56	0.033*
	Step L. var (%)	37.01 ± 9.97	41.84 ± 9.57	33.56 ± 5.38	0.012*	38.30 ± 9.50	42.29 ± 6.25	34.52 ± 7.82	0.010*	-12.57 ± 46.89	-6.29 ± 28.16	-3.91 ± 23.78	0.983
	Step L. med (cm)	40.16 ± 5.76	34.22 ± 4.90	33.18 ± 5.50	<0.001*	39.47 ± 5.55	33.58 ±5.33	30.48 ± 4.32	<0.001*	0.20 ± 17.12	0.02 ± 22.15	6.82 ± 14.73	0.346
	Step L. sym me	0.70 ± 0.10	0.66 ± 0.11	0.71 ± 0.07	0.122	0.71 ± 0.10	0.64 ± 0.11	0.68 ± 0.07	0.044*	-3.18 ± 21.63	-1.27 ± 32.23	4 ± 11.74	0.498
	Step L. sym var (%)	34.87 ± 12.43	38.86 ± 13.25	30.53 ± 8.65	0.084	33.15 ± 13.76	41.50 ± 15.79	36.40 ± 8.37	0.078	-13.79 ± 71.1	-32.45 ± 98.72	-31.96 ± 64.42	0.419
	Step L. sym med	0.75 ± 0.13	0.67 ± 0.16	0.73 ± 0.07	0.054	0.74 ± 0.11	0.66 ± 0.16	0.71 ± 0.11	0.057	-1.45 ± 24.05	-7.99 ± 51.93	2.18 ± 14.55	0.942
	Step W. me (m)	0.21 ± 0.04	0.17 ± 0.03	0.13 ± 0.02	<0.001*	0.21 ± 0.04	0.17 ± 0.04	0.13 ± 0.03	<0.001*	-1.18 ± 19.59	-0.53 ± 26.94	2.69 ± 18.3	0.833
	Step W. var (%)	56.82± 12.33	67.09 ± 13.87	71.76 ± 9.87	<0.001*	59.99 ± 12.72	66.96 ± 12.21	67.12 ± 8.02	0.036*	-12.12 ± 41.22	-2.75 ± 25.18	4.41 ± 19.78	0.172
	Step W. med (m)	0.21 ± 0.06	0.16 ± 0.04	0.12 ± 0.02	<0.001*	0.19 ± 0.04	0.15 ± 0.05	0.12 ± 0.03	<0.001*	0.28 ± 33.81	-2.94 ± 43.82	-0.08 ± 26.58	0.941
	Step H. me (m)	0.12 ±0.02	0.13 ± 0.02	0.13 ± 0.03	0.136	0.12 ± 0.02	0.14 ± 0.02	0.13 ± 0.03	0.081	-4.51 ± 14.83	-6.95 ± 21.55	-1.57 ± 23.73	0.455
	Step H. var (%)	38.65 ± 14.60	46.29 ± 11.20	35.75 ± 9.32	0.001*	32.62 ± 9.74	43.37 ± 11.06	39.87 ± 11.89	0.002*	8.93 ± 33.39	1.26 ± 33.20	-17.96 ± 45.04	0.052
	Step H. med (m)	0.11 ± 0.02	0.12 ± 0.02	0.13 ± 0.02	0.082	0.12 ± .03	0.13 ± 0.02	0.12 ± 0.03	0.491	-7.15 ± 17.57	-7.99 ± 19.6	2.10 ± 24.62	0.096
	Stride L. me (cm)	75.84 ± 10.44	65.91 ± 6.83	64.69 ± 9.08	<0.001*	74.41 ± 9.02	64.49 ± 6.95	57.48 ± 6.83	<0.001*	0.46 ± 15.57	1.37 ± 13.22	10.45 ± 9.82	0.038*
	Stride L. var (%)	27.84 ± 11.09	30.83 ± 12.33	23.74 ± 6.60	0.251	30.66 ± 12.38	29.65 ± 7.64	22.79 ± 8.46	0.028*	-26.86 ± 68.96	-12.52 ± 58.21	-0.39 ± 40.64	0.438
	Stride L. med (cm)	76.46 ± 11.61	65.10 ± 9.50	66.71 ± 10.28	<0.001*	77.38 ± 11.45	65.63 ± 9.77	56.34 ± 6.71	<0.001*	-2.97 ± 18.99	-2.79 ± 21.18	14.45 ± 12.10	0.001*
	Stride L. reg me	0.68 ± 0.12	0.70 ± 0.12	0.77 ± 0.08	0.037*	0.67 ± 0.12	0.69 ± 0.11	0.79 ± 0.08	0.002*	-0.79 ± 24.36	-1.72 ± 25.21	-3.51 ± 15.44	0.626
	Stride L. reg var (%)	23.37 ± 15.17	24.66 ± 14.71	21.98 ± 10.10	0.871	29.21 ± 17.02	30.16 ± 15.94	20.81 ± 14.17	0.081	-10.57± 27.40	-20.30 ± 57.50	-15.31 ± 97.67	0.230
	Stride L. reg med	0.68 ± 0.13	0.71 ± 0.15	0.80 ± 0.09	0.010*	0.66 ± 0.15	0.68 ± 0.14	0.82 ± 0.09	<0.001*	0.10 ± 28.55	0.26 ± 27.59	-4.43 ± 17.87	0.536
**Micro Spatiotemporal**	Step V. me (cm/s)	46.42 ± 8.05	37.17 ± 8.08	32.32 ± 8.24	<0.001*	39.03 ± 8.47	30 ± 5.24	23.48 ± 3.95	<0.001*	15.27 ± 14.18	17.24 ± 15.75	24.65 ± 17.21	0.125
	Step V. var (%)	47.66 ± 9.92	53.47 ± 14.37	47.01 ± 9.39	0.110	56.69 ± 15.63	60.62 ± 10.91	62.89 ± 21.88	0.290	-24.24 ± 44.45	-21.99 ± 43.14	-35.11 ± 43.5	0.390
	Step V. med (cm/s)	46.88 ± 9.48	35.08 ± 9.92	30.82 ± 8.03	<0.001*	37.86 ± 10	27.68 ± 7.08	21.05 ± 4.09	<0.001*	17.44 ± 21.92	16.17 ± 27.15	28.75 ± 18.79	0.127
	Stride V. me (cm/s)	45.52 ± 7.91	35.47 ± 8.51	30.41 ± 7.68	<0.001*	37.17 ± 7.93	28.44 ± 5.28	21.27 ± 4.56	<0.001*	17.75 ± 13.01	17.01 ± 17.83	27.21 ± 19.62	0.098
	Stride V. var (%)	37.77 ± 11.52	38.90 ± 10.05	32.19 ± 5.82	0.077	47.47 ± 11.39	40.90 ± 10.81	38.49 ± 12.55	0.013*	-40.27 ± 61.88	-15.2 ± 54.71	-23.98 ± 46.46	0.203
	Stride V. med (cm/s)	46.35 ± 8.48	34.15 ± 10.06	30.65 ± 7.96	<0.001*	37.15 ± 9.08	27.95 ± 5.30	20.62 ± 4.82	<0.001*	18.94 ± 17.90	13.73 ± 21.74	28.88 ± 23.17	0.055

Data are shown as mean ± standard deviation; *p*-value is reported for comparison between two study groups; *Significant difference at *p*-value <0.05. *HC* = healthy control without cognitive impairment; *MCI* = mild cognitive impairment;*AD* = Alzheimer's Disease; *T* = time; *L* = length; *W* = width; *H* = height; *V* = velocity; *me* = mean; *var* = variability; *sym* = symmetry; *reg* = regularity; *DS* = double support; *SS* = single support.

Table S2. . Comparison between one group vs one group for extracted features from participants in different condition of curve walking.

Feature		*p*-value for single-task curve walking			*p*-value for dual-tasking curve walking			*p*-value dual-tasking cost		
**Type**	**Name**	**MCI vs. HC**	**AD vs. HC**	**AD vs. MCI**	**MCI vs. HC**	**AD vs. HC**	**AD vs. MCI**	**MCI vs. HC**	**AD vs. HC**	**AD vs. MCI**
**Macro**	Duration (s)	<0.001*	<0.001*	<0.001*	<0.001*	<0.001*	<0.001*	0.002*	<0.001*	0.004*
	Velocity (cm/s)	0.007*	<0.001*	0.031*	0.011*	<0.001*	0.002*	0.910	0.083	0.171
	Step number	0.379	<0.001*	<0.001*	0.001*	<0.001*	<0.001*	0.005*	<0.001*	<0.001*
	Cadence	0.018*	<0.001*	0.040*	0.017*	<0.001*	0.043*	0.987	0.660	0.713
**Micro Temporal**	Stance T. me (s)	0.138	<0.001*	<0.001*	0.158	<0.001*	<0.001*	0.767	0.890	0.721
	Stance T. var (%)	<0.001*	0.012*	0.031*	0.052	0.277	0.427*	0.062	0.377	0.242
	Stance T. med (s)	0.384	<0.001*	<0.001*	0.704	<0.001*	<0.001*	0.254	0.907	0.284
	Swing T. me (s)	0.212	0.150	0.017*	0.010*	0.095	0.775	0.135	<0.001*	0.014*
	Swing T. var (%)	0.0184*	0.462	0.103	0.051	0.015*	0.614	0.937	0.377	0.232
	Swing T. med (s)	0.001*	0.322	0.002*	0.008*	0.192	0.488	0.704	0.007*	0.034*
	DS T. me (s)	<0.001*	<0.001*	0.018*	<0.001*	<0.001*	<0.001*	0.491	0.003*	0.021*
	DS T. var (%)	0.072	0.002*	0.382	0.295	0.294	0.885	0.010*	0.002*	0.130
	DS T. med (s)	<0.001*	<0.001*	0.007*	<0.001*	<0.001*	<0.001*	0.032*	<0.001*	0.001*
	SS T. me (s)	0.015*	0.072	0.001*	0.059	0.333	0.023*	0.670	0.264	0.134
	SS T. var (%)	0.896	0.052	0.049*	0.808	0.091	0.071	0.560	0.662	0.815
	SS T. med (s)	0.158	0.002*	<0.001*	0.526	0.130	0.053	0.655	0.159	0.102
	Step T. me (s)	0.012*	<0.001*	0.020*	0.003*	<0.001*	0.008*	0.993	0.617	0.597
	Step T. var (%)	0.018*	0.365	0.281	0.846	0.133	0.223	0.268	0.558	0.164
	Step T. med (s)	0.013*	<0.001*	0.012*	<0.001*	<0.001*	<0.001*	0.089	0.019	0.410
	Step T. sym me	0.207	0.545	0.568	0.526	0.001*	0.014*	0.524	0.007*	0.015*
	Step T. sym var(%)	0.899	0.018*	0.009*	0.163	0.021*	0.597	0.196	0.558	0.102
	Step T. sym med	0.019*	0.004*	0.450	0.069	<0.001*	<0.001*	0.795	<0.001*	<0.001*
	Stride T. me (s)	0.016*	<0.001*	0.024*	0.014*	<0.001*	0.002*	0.630	0.516	0.307
	Stride T. var (%)	0.261	0.884	0.721	0.886	0.070	0.172	0.642	0.388	0.214
	Stride T. med (s)	0.024*	<0.001*	0.031*	<0.001*	<0.001*	0.016*	0.130	0.312	0.796
	Stride T.reg me	0.414	0.486	0.135	0.330	0.496	0.763	0.148	0.835	0.283
	Stride T. reg var (%)	0.729	0.856	0.855	0.742	0.662	0.515	0.833	0.941	0.740
	Stride T. reg med	0.676	0.094	0.039*	0.304	0.681	0.556	0.179	0.476	0.079
**Micro Spatial**	Step L. me (cm)	<0.001*	<0.001*	0.603	<0.001*	<0.001*	0.004*	0.637	0.013*	0.024*
	Step L. var (%)	0.075	0.183	0.002*	0.078	0.161	<0.001*	0.966	0.890	0.772
	Step L. med (cm)	<0.001*	<0.001*	0.516	<0.001*	<0.001*	0.048*	0.973	0.133	0.331
	Step L. sym me	0.114	0.718	0.067	0.021*	0.268	0.217	0.393	0.202	0.931
	Step L. sym var (%)	0.255	0.200	0.025*	0.041*	0.371	0.219	0.431	0.198	0.631
	Step L. sym med	0.030*	0.263	0.115	0.024*	0.363	0.213	0.806	0.565	0.853
	Step W. me (m)	0.002*	<0.001*	0.001*	<0.001*	<0.001*	0.001*	0.918	0.501	0.663
	Step W. var (%)	0.011*	<0.001*	0.097	0.059	0.016*	0.963	0.283	0.075	0.356
	Step W. med (m)	0.022*	<0.001*	0.003*	0.002*	<0.001*	0.024*	0.760	0.969	0.807
	Step H. me (m)	0.069	0.075	0.990	0.025*	0.678	0.143	0.622	0.205	0.356
	Step H. var (%)	0.004*	0.412	0.002*	<0.001*	0.036*	0.284	0.399	0.022*	0.115
	Step H. med (m)	0.114	0.044*	0.363	0.183	0.721	0.515	0.868	0.049*	0.063
	Stride L. me (cm)	<0.001*	<0.001*	0.618	<0.001*	<0.001*	0.002*	0.819	0.019*	0.018*
	Stride L. var (%)	0.393	0.332	0.107	0.725	0.022*	0.008*	0.283	0.292	0.796
	Stride L. med (cm)	<0.001*	0.005*	0.597	<0.001*	<0.001*	0.001*	0.974	<0.001*	0.003*
	Stride L. reg me	0.657	0.011*	0.037*	0.606	0.001*	0.002*	0.890	0.377	0.440
	Stride L. reg var (%)	0.666	0.924	0.508	0.691	0.072	0.033*	0.993	0.108	0.157
	Stride L. reg med	0.369	0.001*	0.035*	0.531	<0.001*	0.001*	0.984	0.292	0.396
**Micro Spatiotemporal**	Step V. me (cm/s)	<0.001*	<0.001*	0.017*	<0.001*	<0.001*	<0.001*	0.628	0.046*	0.151
	Step V. var (%)	0.083	0.824	0.104	0.099	0.462	0.656	0.966	0.198	0.263
	Step V. med (cm/s)	<0.001*	<0.001*	0.172	<0.001*	<0.001*	0.001*	0.848	0.079	0.067
	Stride V. me (cm/s)	<0.001*	<0.001*	0.021*	<0.001*	<0.001*	<0.001*	0.858	0.051	0.082
	Stride V. var (%)	0.704	0.062	0.015*	0.034*	0.006*	0.263	0.083	0.377	0.438
	Stride V. med (cm/s)	<0.001*	<0.001*	0.136	<0.001*	<0.001*	<0.001*	0.412	0.102	0.023*

Data are shown as mean ± standard deviation; *p*-value is reported for comparison between two study groups; *Significant difference at *p*-value <0.05. *HC* = healthy control without cognitive impairment; *MCI* = mild cognitive impairment;*AD* = Alzheimer's Disease; *T* = time; *L* = length; *W* = width; *H* = height; *V* = velocity; *me* = mean; *var* = variability; *sym* = symmetry; *reg* = regularity; *DS* = double support; *SS* = single support.

all dual-task-cost markers, for which the family of tests is smallest and the BH adjustment correspondingly most severe.

[B1] Benjamini Y, Hochberg Y. Controlling the false discovery rate: a practical and powerful approach to multiple testing. *Journal of the Royal Statistical Society: Series B (Methodological)*. 1995;57(1):289–300.

[B2] Storey JD, Tibshirani R. Statistical significance for genomewide studies. *Proceedings of the National Academy of Sciences*. 2003;100(16):9440–9445.

S3 FOLD-LEVEL MEAN $\pm$ STANDARD DEVIATION OF CLASSIFICATION PERFORMANCE METRICS

Table S7 reports the fold-level mean $\pm$ standard deviation across the five outer cross-validation folds for each combination of classifier input set (single-task gait markers, dual-task gait markers, and their combination) and group contrast (CI vs. HC, and mild AD vs. MCI). The metrics reported are accuracy, sensitivity, specificity, precision (positive predictive value, PPV), F-score, and negative predictive value (NPV). These per-fold summaries quantify the fold-to-fold stability of the performance estimates reported in Table 3 of the main article.

S4 POST-HOC POWER ANALYSIS FOR THE HEADLINE GROUP CONTRASTS

To quantify the statistical power of the headline group contrasts, we performed a post-hoc analysis using participant-level data. For each gait marker $\times$ condition combination, we computed Cohen's d for the CI-vs-HC contrast (with the MCI and AD groups pooled into a single CI group of $n = 43$, against HC $n = 30$) and for the AD-vs-MCI contrast ($n = 18$ vs. $n = 25$). Achieved power at $\alpha = 0.05$ (two-sided) was computed from the normal approximation with non-centrality parameter $\mathrm{ncp} = d \cdot \sqrt{n_1 n_2/(n_1 + n_2)}$.

Across the 54 marker $\times$ condition entries reaching 3-group $p < 0.001$, the mean Cohen's d was 1.16 for CI vs. HC and 1.01 for AD vs. MCI, with mean achieved power of 0.94 and 0.72 respectively. The AD subgroup ($n = 18$) is the binding sample-size constraint, although several individual markers for the AD-vs-MCI contrast achieve power ≥ 0.95 (Table S9). Effect sizes are computed from participant-level data rather than from the rounded summary statistics of Table S1, because rounding to two decimal places materially distorts d for markers of small numerical magnitude such as step width in metres. The top

15 markers per contrast are listed in Tables S8 and S9; the full list of markers is available from the corresponding author upon reasonable request.

S5 CONFIDENCE INTERVALS FOR THE REPORTED CLASSIFICATION ACCURACIES

Because the reviewer asked specifically for confidence intervals around the headline classification performance, Table S10 reports Wilson score 95% confidence intervals for the cross-validated accuracy of each model. These are computed from the number of correctly classified participants out of the total cohort available for each contrast ($n = 73$ for CI vs. HC; $n = 43$ for AD vs. MCI) and therefore reflect the finite size of the cohort rather than fold-to-fold variability. The intervals are correspondingly wide, particularly for the AD-vs-MCI contrast, and they indicate the precision attainable at the present sample size.

S6 PERMUTATION IMPORTANCE OF THE SELECTED GAIT MARKERS

The effect-size ranking reported in Section S4 is univariate: it measures how strongly each marker separates the groups on its own, but says nothing about how the trained classifier uses that marker. We therefore also computed permutation importance for the combined single-task + dual-task SVM models reported in Table 3 of the main article.

Permutation importance was computed strictly on held-out data. For each cross-validation fold, the complete pipeline—within-fold marker selection, standardisation, and SVM hyperparameter grid search—was fitted on the training partition only. Each retained marker in the held-out partition was then randomly permuted 30 times, and the resulting mean decrease in balanced accuracy was recorded as that marker's importance for that fold. Because the fold-level test partitions are small ($n \approx 15$ for CI vs. HC and $n \approx 9$ for AD vs. MCI), the entire 5-fold procedure was repeated over 10 random fold assignments, giving 50 independent training/test splits. Reported importance is the mean across the folds in which a marker was retained by the selection step. We also report each marker's selection frequency, that is, the percentage of the 50 folds in which it survived selection, so that markers used consistently across folds can be distinguished from those retained only occasionally.

The analysis characterises the models already reported; the classification pipeline, the marker pool, and the accuracies in Table 3 of the main article are unchanged. Tables S11 and S12 list the 15 highest-ranked markers for each headline contrast.

The resulting ranking agrees closely with the univariate effect-size ranking in Tables S8 and S9. Duration, step number, double support time, step width, and stride velocity appear near the top of both, indicating that the markers driving the classifier are also those showing the largest group-level separation. Dual-task and dual-task-cost markers occupy 11 of the top 15 positions for the CI-vs-HC contrast, which corroborates the finding that dual-tasking adds discriminative information beyond single-task curved walking using a criterion derived from the classifier rather than from univariate testing. For the AD-vs-MCI contrast, the two highest-ranked markers are dual-task step number and single-task duration, both macro-level pacing markers, consistent with the progressive slowing and increase in step count reported in Section 3.1 of the main article.

Table S3. FDR audit of correlation-pruning–selected gait markers for CI vs. HC under single-task curved walking. Raw p-values are from Table 2 of the main article. Benjamini–Hochberg adjusted q-values are reported for the (contrast × condition) family; ✓ indicates survival at the stated threshold. Of the 20 selected markers, 15 survive at $q \leq 0.05$ and 20 at $q \leq 0.10$.

Marker	Condition	Raw p	BH q	$q \leq 0.05$	$q \leq 0.10$
Duration	ST	<0.001	0.002	✓	✓
Velocity	ST	<0.001	0.002	✓	✓
Step Number	ST	0.009	0.018	✓	✓
Stance Time Mean	ST	<0.001	0.002	✓	✓
Stance Time Variability	ST	<0.001	0.002	✓	✓
Double Support Time Mean	ST	<0.001	0.002	✓	✓
Double Support Time Variability	ST	0.005	0.010	✓	✓
Double Support Time Median	ST	<0.001	0.002	✓	✓
Step Time Symmetry Median	ST	0.003	0.006	✓	✓
Step Time Variability	ST	0.039	0.068	–	✓
Step Time Median	ST	<0.001	0.002	✓	✓
Step Length Median	ST	<0.001	0.002	✓	✓
Step Length Symmetry Median	ST	0.041	0.069	–	✓
Step Width Variability	ST	<0.001	0.002	✓	✓
Step Width Median	ST	<0.001	0.002	✓	✓
Step Height Mean	ST	0.046	0.075	–	✓
Step Height Median	ST	0.034	0.063	–	✓
Stride Length Mean	ST	<0.001	0.002	✓	✓
Stride Length Regularity Median	ST	0.029	0.056	–	✓
Stride Velocity Median	ST	<0.001	0.002	✓	✓

Table S4. FDR audit of correlation-pruning–selected gait markers for AD vs. MCI under single-task curved walking. Raw p-values are from Table 2 of the main article. Benjamini–Hochberg adjusted q-values are reported for the (contrast × condition) family; ✓ indicates survival at the stated threshold. Of the 19 selected markers, 11 survive at $q \leq 0.05$ and 19 at $q \leq 0.10$.

Marker	Condition	Raw p	BH q	$q \leq 0.05$	$q \leq 0.10$
Duration	ST	<0.001	0.007	✓	✓
Step Number	ST	<0.001	0.007	✓	✓
Stance Time Mean	ST	<0.001	0.007	✓	✓
Stance Time Variability	ST	0.031	0.067	–	✓
Swing Time Median	ST	0.002	0.009	✓	✓
Double Support Time Mean	ST	0.018	0.052	–	✓
Double Support Time Median	ST	0.007	0.028	✓	✓
Single Support Time Variability	ST	0.049	0.088	–	✓
Single Support Time Median	ST	<0.001	0.007	✓	✓
Step Time Symmetry Variability	ST	0.009	0.033	✓	✓
Stride Time Regularity Median	ST	0.039	0.074	–	✓
Step Length Variability	ST	0.002	0.009	✓	✓
Step Length Symmetry Variability	ST	0.025	0.059	–	✓
Step Width Mean	ST	0.001	0.007	✓	✓
Step Width Median	ST	0.003	0.013	✓	✓
Step Height Variability	ST	0.002	0.009	✓	✓
Stride Length Regularity Median	ST	0.035	0.073	–	✓
Step Velocity Mean	ST	0.017	0.052	–	✓
Stride Velocity Variability	ST	0.015	0.052	–	✓

Table S5. FDR audit of correlation-pruning–selected gait markers for CI vs. HC under dual-task curved walking. Raw p-values are from Table 2 of the main article. Benjamini–Hochberg adjusted q-values are reported for the (contrast $\times$ condition) family; ✓ indicates survival at the stated threshold. Of the 27 selected markers, 23 survive at $q \leq 0.05$ and 26 at $q \leq 0.10$.

Marker	Condition	Raw p	BH q	$q \leq 0.05$	$q \leq 0.10$
Duration	DT	<0.001	0.002	✓	✓
Swing Time Mean	DT	0.009	0.018	✓	✓
Swing Time Variability	DT	0.005	0.011	✓	✓
Double Support Time Median	DT	<0.001	0.002	✓	✓
Step Time Mean	DT	<0.001	0.002	✓	✓
Step Time Median	DT	<0.001	0.002	✓	✓
Step Time Symmetry Mean	DT	0.032	0.050	–	✓
Step Time Symmetry Variability	DT	0.049	0.073	–	✓
Step Time Symmetry Median	DT	<0.001	0.002	✓	✓
Duration Cost	DTC	<0.001	0.012	✓	✓
Step Number Cost	DTC	<0.001	0.012	✓	✓
Swing Time Mean Cost	DTC	0.003	0.031	✓	✓
Double Support Time Variability Cost	DTC	<0.001	0.012	✓	✓
Double Support Time Median Cost	DTC	<0.001	0.012	✓	✓
Step Time Median Cost	DTC	0.018	0.156	–	–
Step Length Mean	DT	<0.001	0.002	✓	✓
Step Length Median	DT	<0.001	0.002	✓	✓
Step Width Mean	DT	<0.001	0.002	✓	✓
Step Width Variability	DT	0.011	0.021	✓	✓
Step Width Median	DT	<0.001	0.002	✓	✓
Step Height Variability	DT	<0.001	0.002	✓	✓
Step Length symmetry Mean	DT	0.029	0.047	✓	✓
Step Length symmetry Median	DT	0.048	0.073	–	✓
Stride Length Median	DT	<0.001	0.002	✓	✓
Stride Length Regularity Median	DT	0.021	0.038	✓	✓
Stride Velocity Mean	DT	<0.001	0.002	✓	✓
Stride Velocity Variability	DT	0.007	0.015	✓	✓

Table S6. FDR audit of correlation-pruning–selected gait markers for AD vs. MCI under dual-task curved walking. Raw p-values are from Table 2 of the main article. Benjamini–Hochberg adjusted q-values are reported for the (contrast $\times$ condition) family; ✓ indicates survival at the stated threshold. Of the 30 selected markers, 23 survive at $q \leq 0.05$ and 25 at $q \leq 0.10$.

Marker	Condition	Raw p	BH q	$q \leq 0.05$	$q \leq 0.10$
Duration	DT	<0.001	0.003	✓	✓
Velocity	DT	0.002	0.005	✓	✓
Step Number	DT	<0.001	0.003	✓	✓
Double Support Time Median	DT	<0.001	0.003	✓	✓
Single Support Time Mean	DT	0.023	0.048	✓	✓
Step Time Symmetry Mean	DT	0.014	0.032	✓	✓
Step Time Symmetry Median	DT	<0.001	0.003	✓	✓
Stride Time Mean	DT	0.002	0.005	✓	✓
Step Length Variability	DT	<0.001	0.003	✓	✓
step Length Median	DT	0.048	0.086	–	✓
Step Width Mean	DT	0.001	0.003	✓	✓
Step Width Median	DT	0.024	0.048	✓	✓
Stride Length Mean	DT	0.002	0.005	✓	✓
Stride Length Variability	DT	0.008	0.019	✓	✓
Stride Length Median	DT	0.001	0.003	✓	✓
Stride Length Regularity Variability	DT	0.033	0.064	–	✓
Stride Length Regularity Median	DT	0.001	0.003	✓	✓
Step Velocity Mean	DT	<0.001	0.003	✓	✓
Step Velocity Median	DT	0.001	0.003	✓	✓
Stride Velocity Median	DT	<0.001	0.003	✓	✓
Duration Cost	DTC	0.004	0.042	✓	✓
Step Number Cost	DTC	<0.001	0.017	✓	✓
Swing Time Mean Cost	DTC	0.014	0.111	–	–
Swing Time Median Cost	DTC	0.034	0.136	–	–
Double Support Time Median Cost	DTC	0.001	0.017	✓	✓
Step Time Symmetry Mean Cost	DTC	0.015	0.111	–	–
Step Time Symmetry Median Cost	DTC	<0.001	0.017	✓	✓
Step Length Mean Cost	DTC	0.024	0.113	–	–
Stride Length Median Cost	DTC	0.003	0.039	✓	✓
Stride Velocity Median Cost	DTC	0.023	0.113	–	–

Table S7. Fold-level mean ± standard deviation across five-fold subject-wise cross-validation results of machine learning model for discrimination of CI vs. HC, and Mild AD vs. MCI using single-task, dual-task gait markers and their combination.

Groups	Accuracy	Sensitivity	Specificity	Precision (PPV)	F-score	NPV
**A. Single-task gait markers**						
CI vs. HC	79.45±6.07	81.39±16.18	76.67±14.91	83.33±9.28	82.35 ±8.68	74.19±9.60
Mild AD vs. MCI	88.37±6.48	84.44±11.18	84.00±8.94	80.95±11.18	87.18±7.82	88.23±7.45
Overall accuracy = 83.91±6.27%						
**B. Dual-task gait markers**						
CI vs. HC	84.93±8.66	83.72±12.73	86.67±9.13	90.00±6.60	86.75±7.84	78.79±15.07
Mild AD vs. MCI	90.69±6.84	88.89±7.90	92.00±8.94	88.89±11.18	88.89±9.60	92.00±7.45
Overall accuracy = 87.81±7.75%						
**C. Combining dual-task and single-tasks gait markers**						
CI vs. HC	89.04±6.37	88.37±7.30	90.00±9.12	92.68±7.00	90.48±8.84	84.37±8.39
Mild AD vs. MCI	93.02±5.59	94.44±5.72	93.00±8.94	89.47±8.84	91.90±11.18	95.87±5.72
Overall accuracy = 91.03±5.98%						

Data are shown as mean ± standard deviation; *CI* = cognitive impairment; *HC* = healthy control without cognitive impairment; *MCI* = mild cognitive impairment;*AD* = Alzheimer's disease; *PPV* = positive predictive value; *NPV* = negative predictive value.

Table S8. Top 15 gait markers ranked by Cohen's d for the CI vs. HC contrast, with achieved statistical power at $\alpha = 0.05$. Computed from participant-level data. CI denotes the pooled MCI+AD group ($n = 43$); HC denotes the healthy-control group ($n = 30$).

Gait marker	Condition	Cohen's d	Achieved power
Stride V. Mean	DT	1.699	1.000
Step V. Mean	DT	1.687	1.000
Double Support T. Mean	ST	1.645	1.000
Duration	DT	1.644	1.000
Stride V. Med.	DT	1.630	1.000
Step L. Mean	DT	1.592	1.000
Stride L. Mean	DT	1.560	1.000
Step T. Med.	DT	1.532	1.000
Stride V. Med.	ST	1.523	1.000
Step V. Med.	DT	1.520	1.000
Stride L. Med.	DT	1.496	1.000
Stride V. Mean	ST	1.475	1.000
Step W. Mean	DT	1.459	1.000
Step V. Med.	ST	1.448	1.000
Step Number	DT	1.402	1.000

V. = velocity; *T.* = time; *L.* = length; *W.* = width; *H.* = height; *Var.* = variability; *Reg.* = regularity; *Med.* = median; *Sym.* = symmetry; *ST* = single-task; *DT* = dual-task; *DTC* = dual-task cost.

Table S9. Top 15 gait markers ranked by Cohen's d for the AD vs. MCI contrast, with achieved statistical power at $\alpha = 0.05$. Computed from participant-level data. AD $n = 18$; MCI $n = 25$.

Gait marker	Condition	Cohen's d	Achieved power
Step Number	DT	2.764	1.000
Duration	DT	2.679	1.000
Stance T. Mean	ST	2.044	1.000
Stance T. Med.	ST	1.979	1.000
Duration	ST	1.941	1.000
Double Support T. Med.	DT	1.891	1.000
Double Support T. Mean	DT	1.581	0.999
Stance T. Mean	DT	1.570	0.999
Stride V. Med.	DT	1.435	0.996
Stride V. Mean	DT	1.434	0.996
Step Number	ST	1.419	0.996
Step T. Symmetry Med.	DT	1.391	0.994
Step V. Mean	DT	1.374	0.993
Stance T. Med.	DT	1.274	0.985
Step T. Med.	DT	1.165	0.965

V. = velocity; *T.* = time; *L.* = length; *W.* = width; *H.* = height; *Var.* = variability; *Reg.* = regularity; *Med.* = median; *Sym.* = symmetry; *ST* = single-task; *DT* = dual-task; *DTC* = dual-task cost.

Table S10. Cross-validated classification accuracy with Wilson score 95% confidence intervals.

Marker set	Contrast	Accuracy (%)	95% CI (%)
Single-task	CI vs. HC	79.45	68.8–87.1
Single-task	Mild AD vs. MCI	88.37	75.5–94.9
Dual-task	CI vs. HC	84.93	75.0–91.4
Dual-task	Mild AD vs. MCI	90.69	78.4–96.3
Combined ST+DT	CI vs. HC	89.04	79.8–94.3
Combined ST+DT	Mild AD vs. MCI	93.02	81.4–97.6

Table S11. Top 15 gait markers ranked by permutation importance for the CI vs. HC contrast, computed on the held-out folds of the combined single-task + dual-task SVM model and restricted to markers retained in at least half of the folds. CI denotes the pooled MCI+AD group ($n = 43$); HC denotes the healthy-control group ($n = 30$).

Rank	Gait marker	Condition	Permutation importance	Selection frequency
1	Stride V. Var.	DT	0.0280	90%
2	Step T. Symmetry Med.	ST	0.0223	94%
3	Step W. Mean	DT	0.0157	100%
4	Double Support T. Variability	DTC	0.0145	100%
5	Step H. Var.	DT	0.0133	100%
6	Duration	DT	0.0120	100%
7	Stride L. Reg. Med.	ST	0.0119	54%
8	Step Number	DT	0.0117	100%
9	Double Support T. Mean	ST	0.0116	100%
10	Double Support T. Mean	DT	0.0114	98%
11	Stance T. Variability	ST	0.0107	100%
12	Double Support T. Med.	DT	0.0106	100%
13	Stride T. Med.	DT	0.0101	100%
14	Step L. Mean	DT	0.0101	96%
15	Double Support T. Med.	DTC	0.0099	100%

Permutation importance is the mean decrease in balanced accuracy on the held-out fold when the marker is randomly permuted (30 permutations per fold), averaged over the folds in which the marker was retained by the within-fold selection procedure. Selection frequency is the percentage of the 50 training folds (10 repeats $\times$ 5 folds) in which the marker survived selection. To keep importances comparable across rows, the ranking is restricted to markers retained in at least half of the folds; markers selected only occasionally are averaged over too few folds to be estimated reliably. *T.* = time; *L.* = length; *W.* = width; *H.* = height; *V.* = velocity; *Var.* = variability; *Reg.* = regularity; *Med.* = median; *ST* = single-task; *DT* = dual-task; *DTC* = dual-task cost.

Table S12. Top 15 gait markers ranked by permutation importance for the AD vs. MCI contrast, computed on the held-out folds of the combined single-task + dual-task SVM model and restricted to markers retained in at least half of the folds. AD $n = 18$; MCI $n = 25$.

Rank	Gait marker	Condition	Permutation importance	Selection frequency
1	Step Number	DT	0.0214	100%
2	Duration	ST	0.0143	100%
3	Step Number	ST	0.0132	100%
4	Stance T. Mean	ST	0.0130	92%
5	Double Support T. Med.	DTC	0.0103	100%
6	Double Support T. Med.	DT	0.0097	100%
7	Swing T. Mean	ST	0.0084	66%
8	Stance T. Med.	ST	0.0082	76%
9	Swing T. Med.	ST	0.0080	96%
10	Stance T. Med.	DT	0.0076	84%
11	Stride V. Med.	DT	0.0072	100%
12	Stride L. Reg. Med.	DT	0.0068	100%
13	Duration	DT	0.0063	100%
14	Single Support T. Med.	ST	0.0058	90%
15	Stance T. Mean	DT	0.0051	90%

Permutation importance is the mean decrease in balanced accuracy on the held-out fold when the marker is randomly permuted (30 permutations per fold), averaged over the folds in which the marker was retained by the within-fold selection procedure. Selection frequency is the percentage of the 50 training folds (10 repeats $\times$ 5 folds) in which the marker survived selection. To keep importances comparable across rows, the ranking is restricted to markers retained in at least half of the folds; markers selected only occasionally are averaged over too few folds to be estimated reliably. *T.* = time; *L.* = length; *W.* = width; *H.* = height; *V.* = velocity; *Var.* = variability; *Reg.* = regularity; *Med.* = median; *ST* = single-task; *DT* = dual-task; *DTC* = dual-task cost.
